# Correction: Biosynthesis of Firefly Luciferin in Adult Lantern: Decarboxylation of L-Cysteine is a Key Step for Benzothiazole Ring Formation in Firefly Luciferin Synthesis

**DOI:** 10.1371/journal.pone.0095063

**Published:** 2014-04-07

**Authors:** The PLOS ONE Staff

## Notice of Republication

This article was republished on March 19, 2014, to replace an incorrectly published version. The publisher apologizes for these errors.

## Supporting Information

File S1 **Originally published, uncorrected article.**
(PDF)

File S2 **Republished corrected article.**
(PDF)
