# Supplementary figures and images for: Biosynthesis of Firefly Luciferin in Adult Lantern: Decarboxylation of ʟ-Cysteine is a Key Step for Benzothiazole Ring Formation in Firefly Luciferin Synthesis
Source: PLoS One. 2013 Dec 31;8(12):e84023. doi: 10.1371/journal.pone.0084023 (PMC3877152; doi:10.1371/journal.pone.0084023)

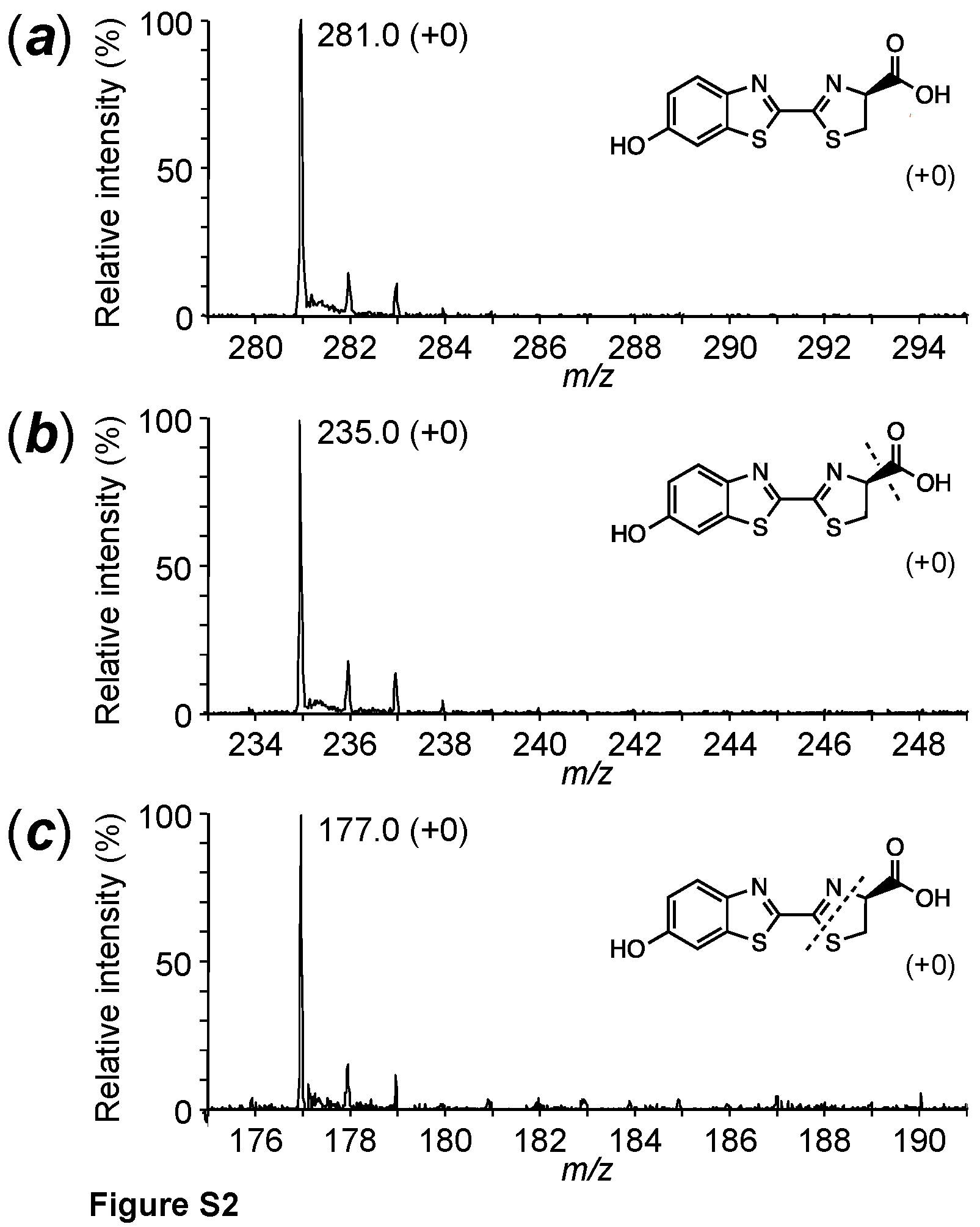

Supplement: Figure S2 — Mass spectra of synthetic ᴅ-firefly luciferin. (a), the parent ion of firefly luciferin; (b) and (c), the fragment ions of firefly luciferin. (TIF) [file pone.0084023.s002.tif]

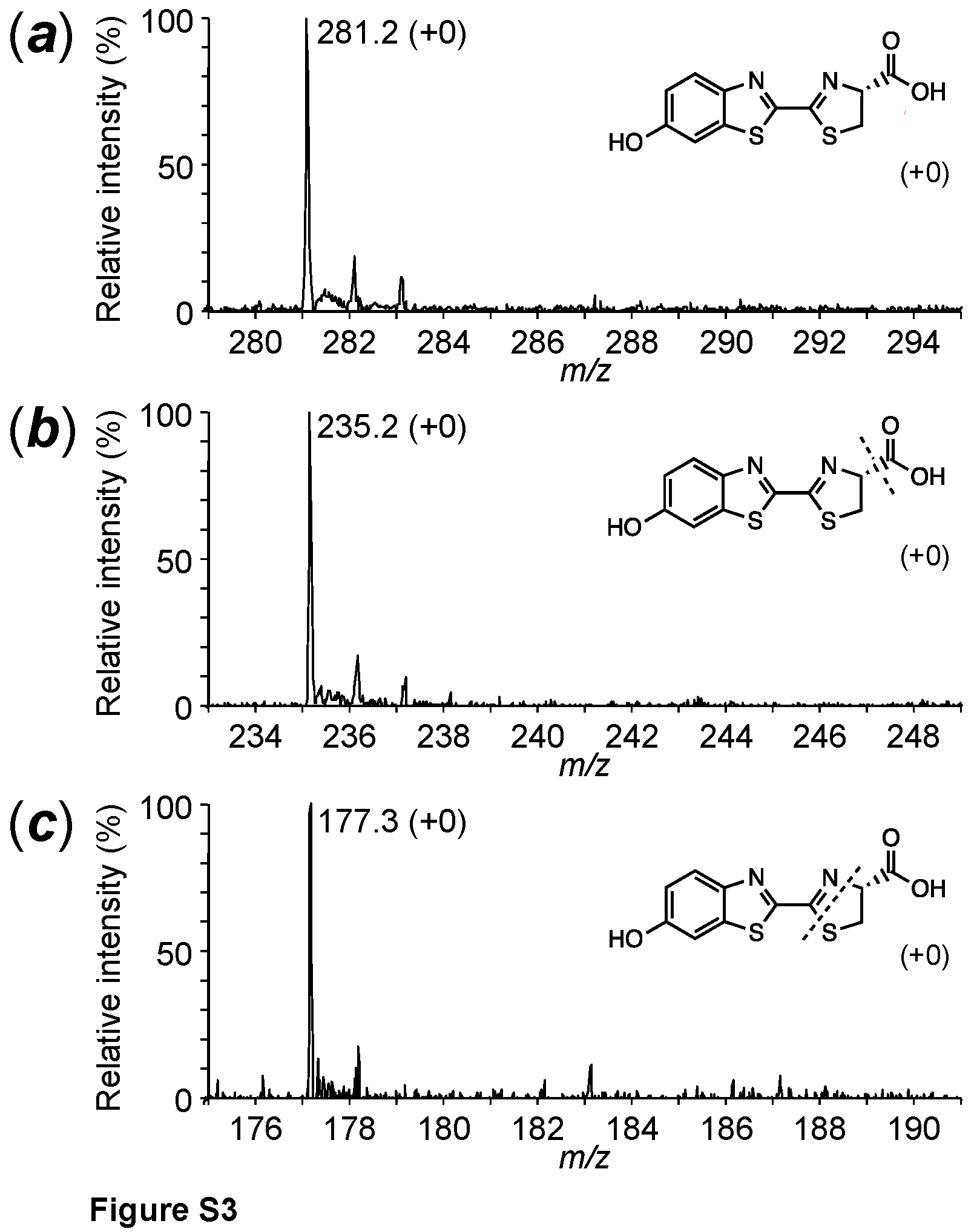

Supplement: Figure S3 — Mass spectra of synthetic ʟ-firefly luciferin. (a), the parent ion of firefly luciferin; (b) and (c), the fragment ions of firefly luciferin. (TIF) [file pone.0084023.s003.tif]

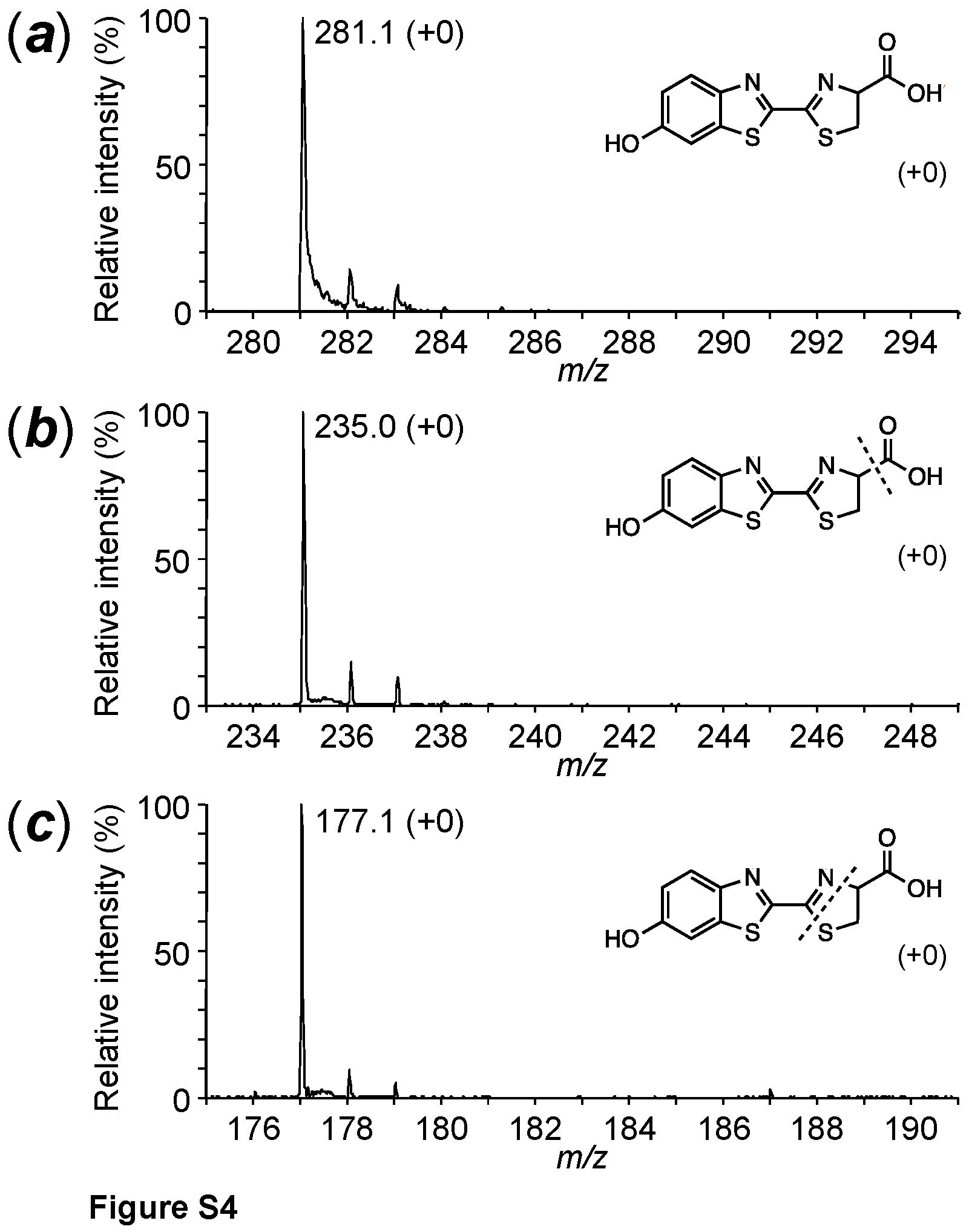

Supplement: Figure S4 — Mass spectra of firefly luciferin extracted from an adult lantern of L. lateralis without injecting the labeled compounds. (a), the parent ion of firefly luciferin; (b) and (c), the fragment ions of firefly luciferin. (TIF) [file pone.0084023.s004.tif]

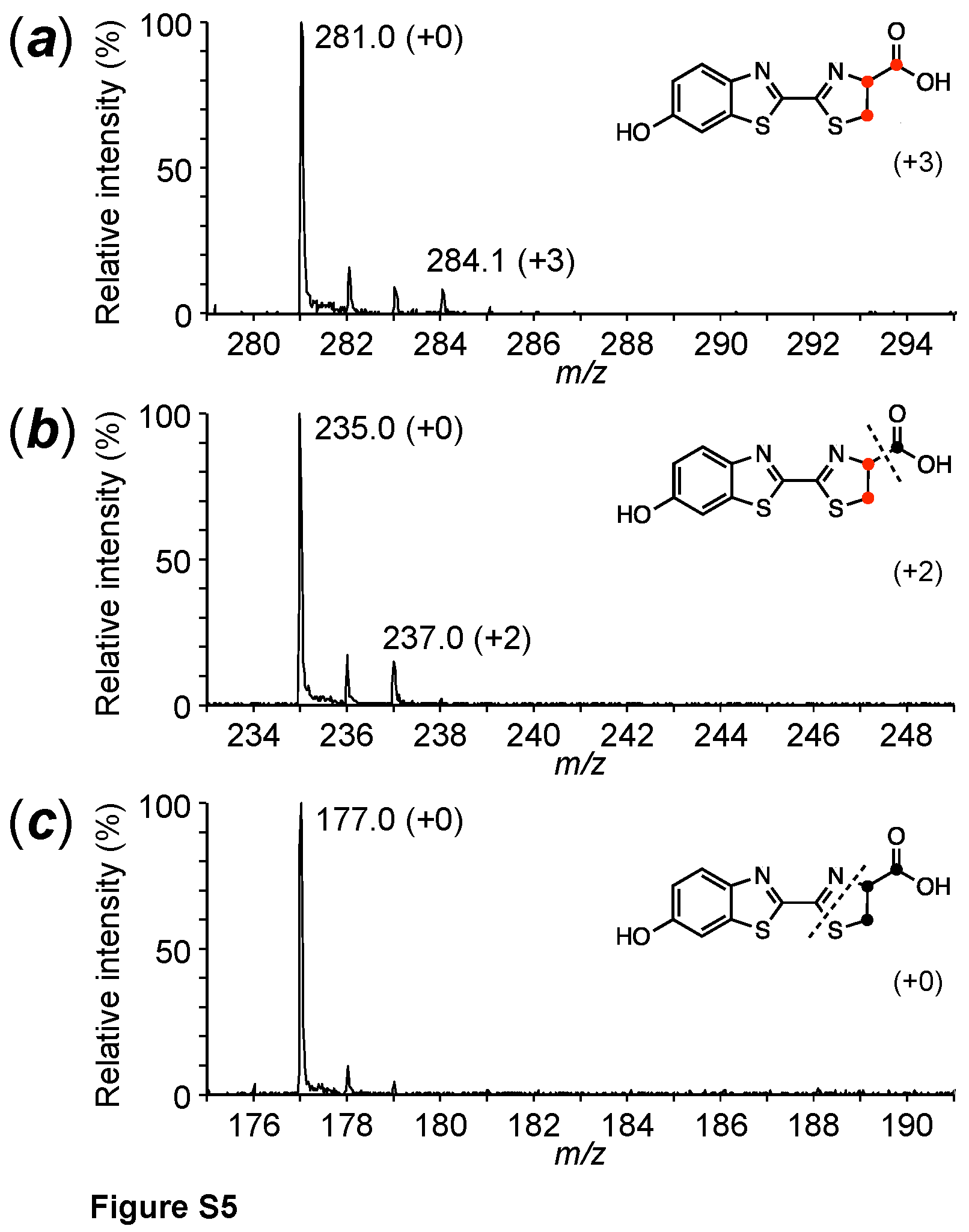

Supplement: Figure S5 — Injecting of ʟ-Cys[U-13C3] into an adult lantern of L. lateralis. (a), the parent ion of firefly luciferin; (b) and (c), the fragment ions of firefly luciferin. (TIF) [file pone.0084023.s005.tif]

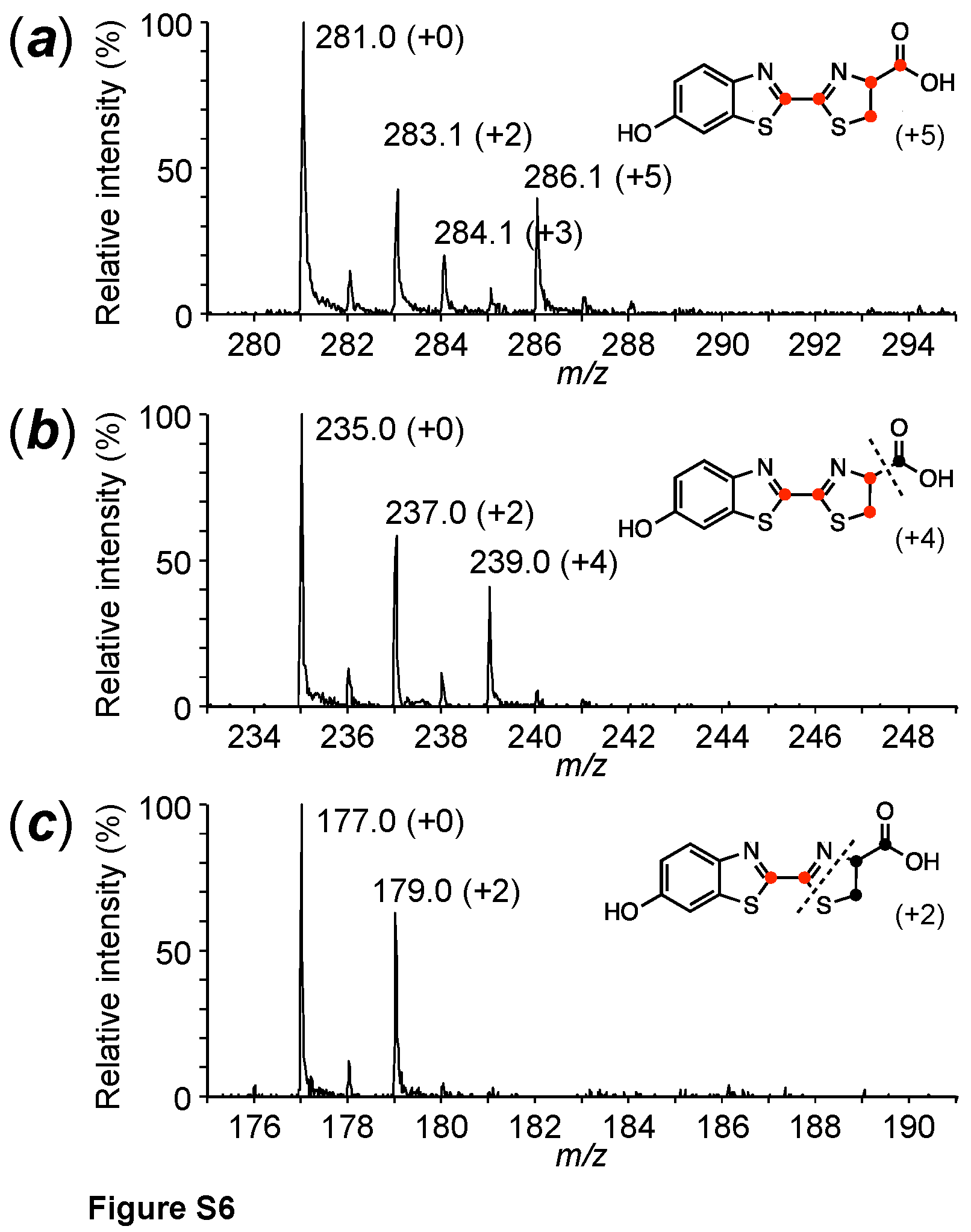

Supplement: Figure S6 — Injecting of ʟ-Cys[U-13C3] and p -benzoquinone into an adult lantern of L. lateralis. (a), the parent ion of firefly luciferin; (b) and (c), the fragment ions of firefly luciferin. (TIF) [file pone.0084023.s006.tif]

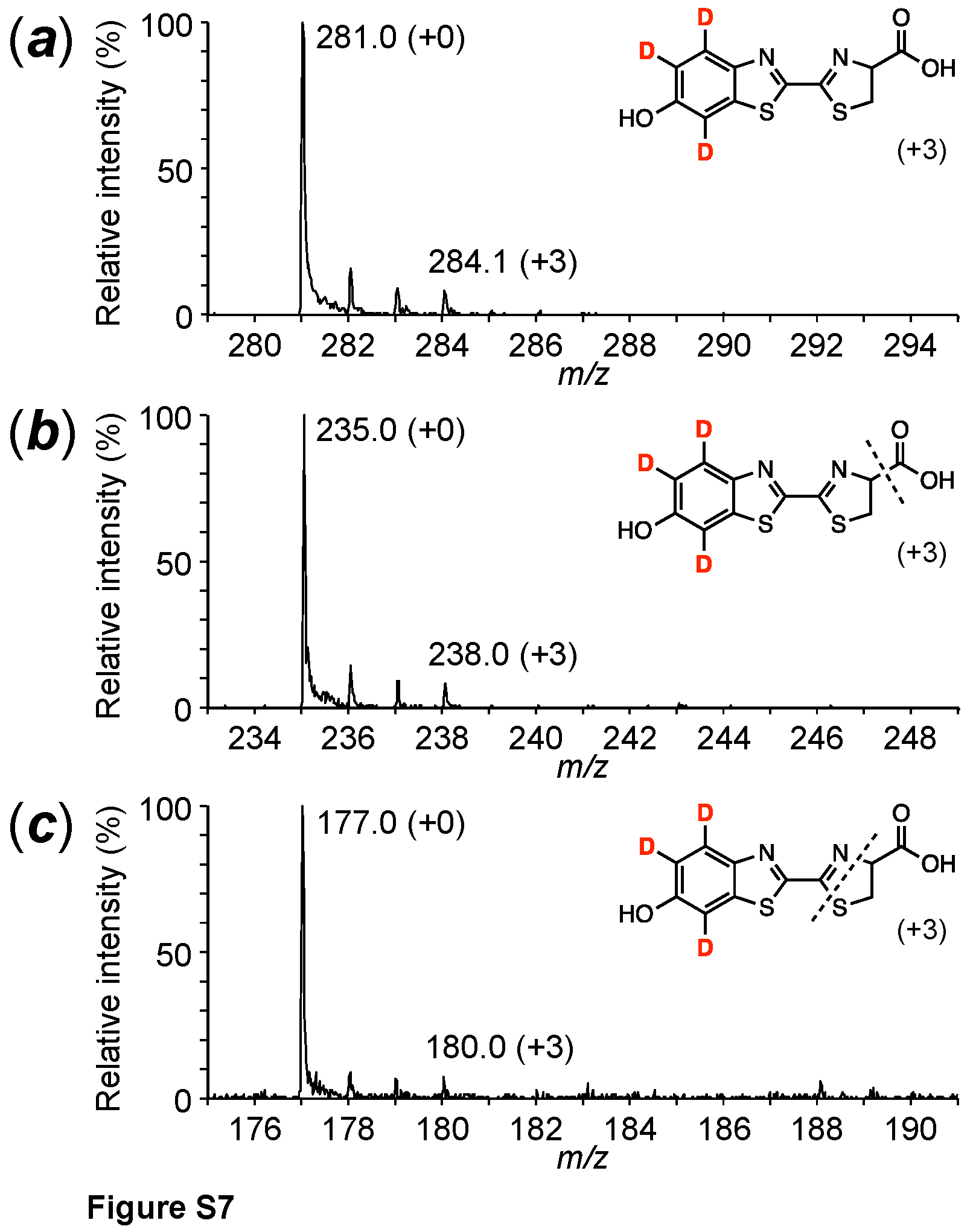

Supplement: Figure S7 — Injecting of 1,4-[D6]-hydroquinone into an adult lantern of L. lateralis. (a), the parent ion of firefly luciferin; (b) and (c), the fragment ions of firefly luciferin. (TIF) [file pone.0084023.s007.tif]

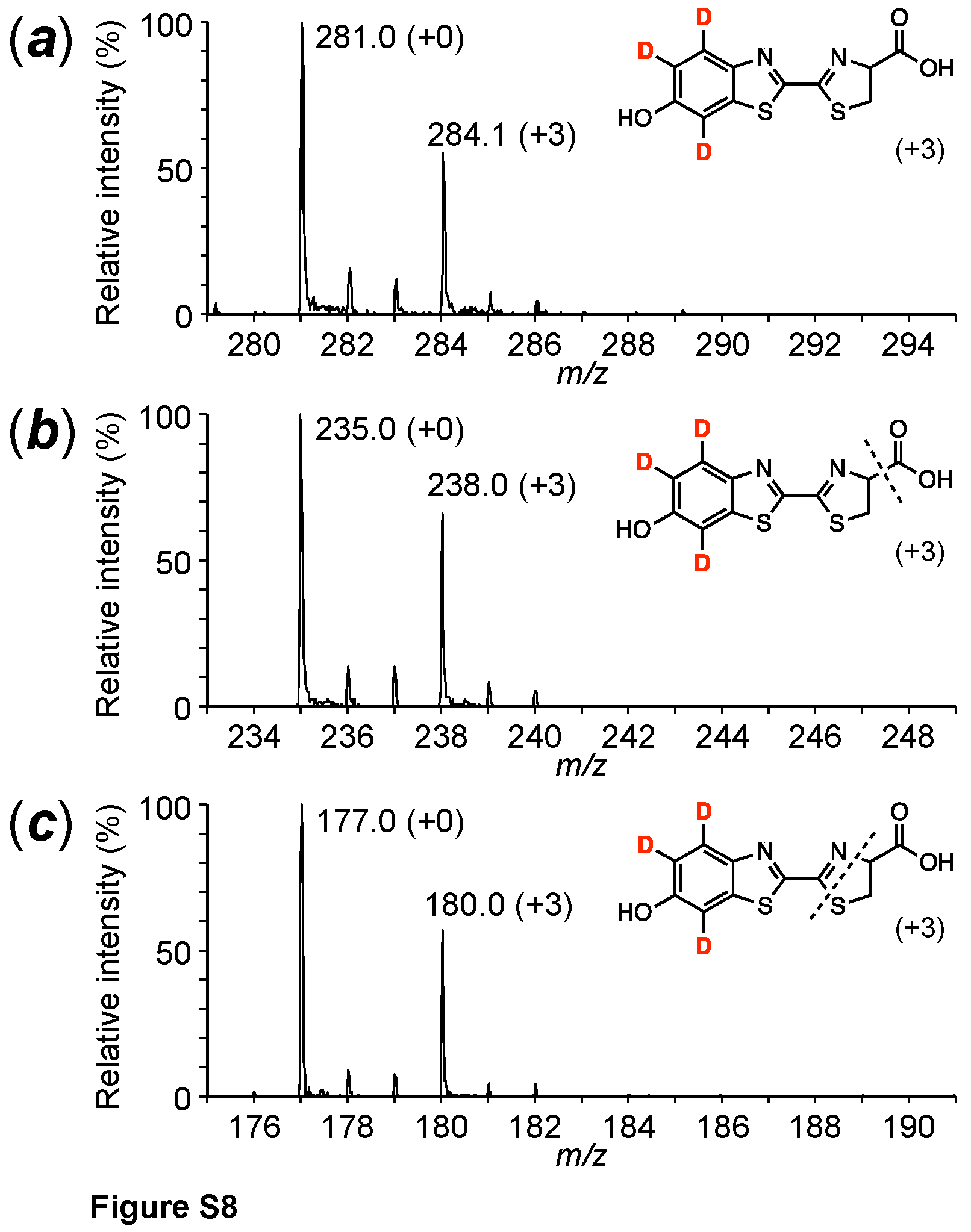

Supplement: Figure S8 — Injecting of 1,4-[D6]-hydroquinone and ʟ-Cys into an adult lantern of L. lateralis. (a), the parent ion of firefly luciferin; (b) and (c), the fragment ions of firefly luciferin. (TIF) [file pone.0084023.s008.tif]

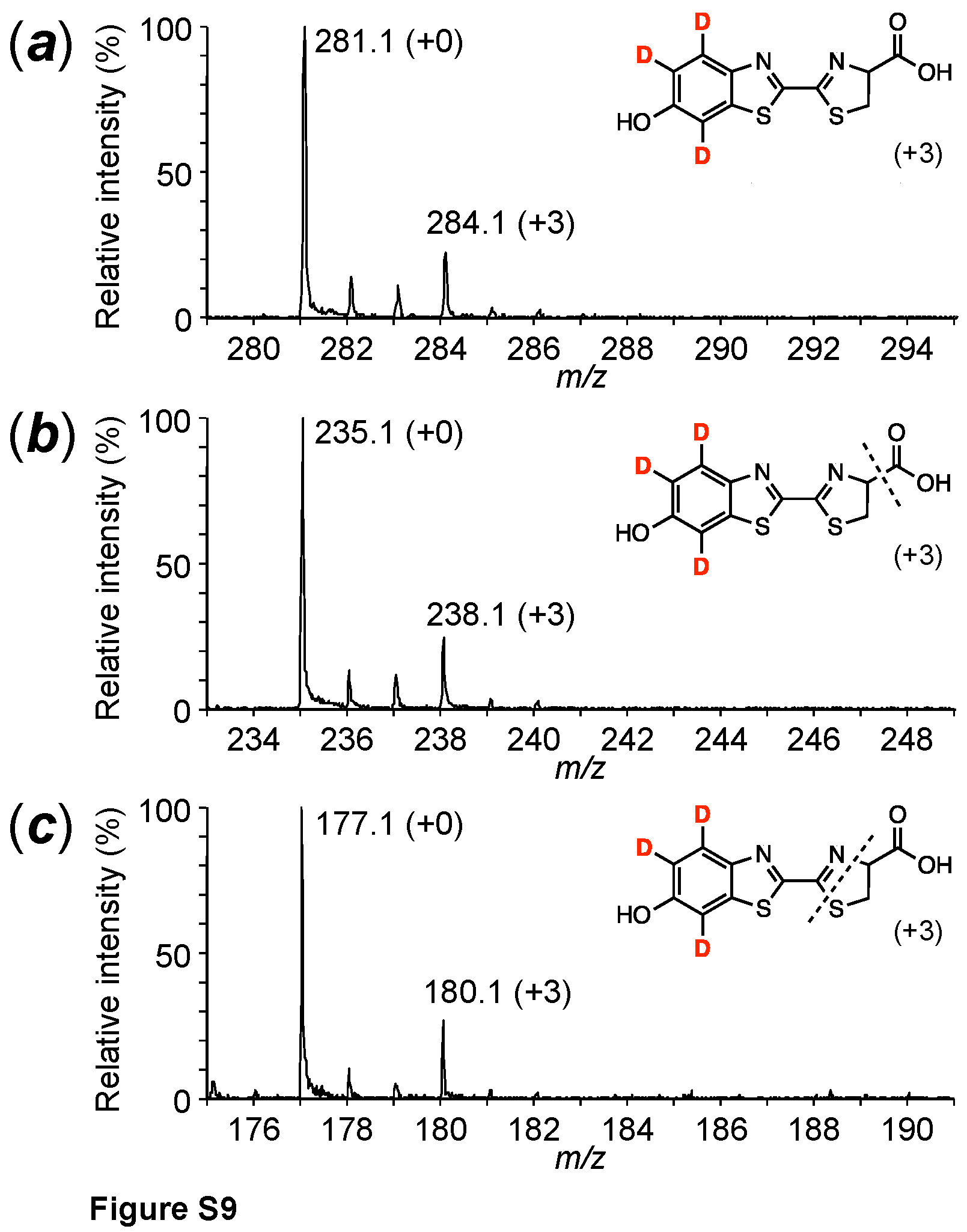

Supplement: Figure S9 — Injecting of p -[D4]-benzoquinone into an adult lantern of L. lateralis. (a), the parent ion of firefly luciferin; (b) and (c), the fragment ions of firefly luciferin. (TIF) [file pone.0084023.s009.tif]

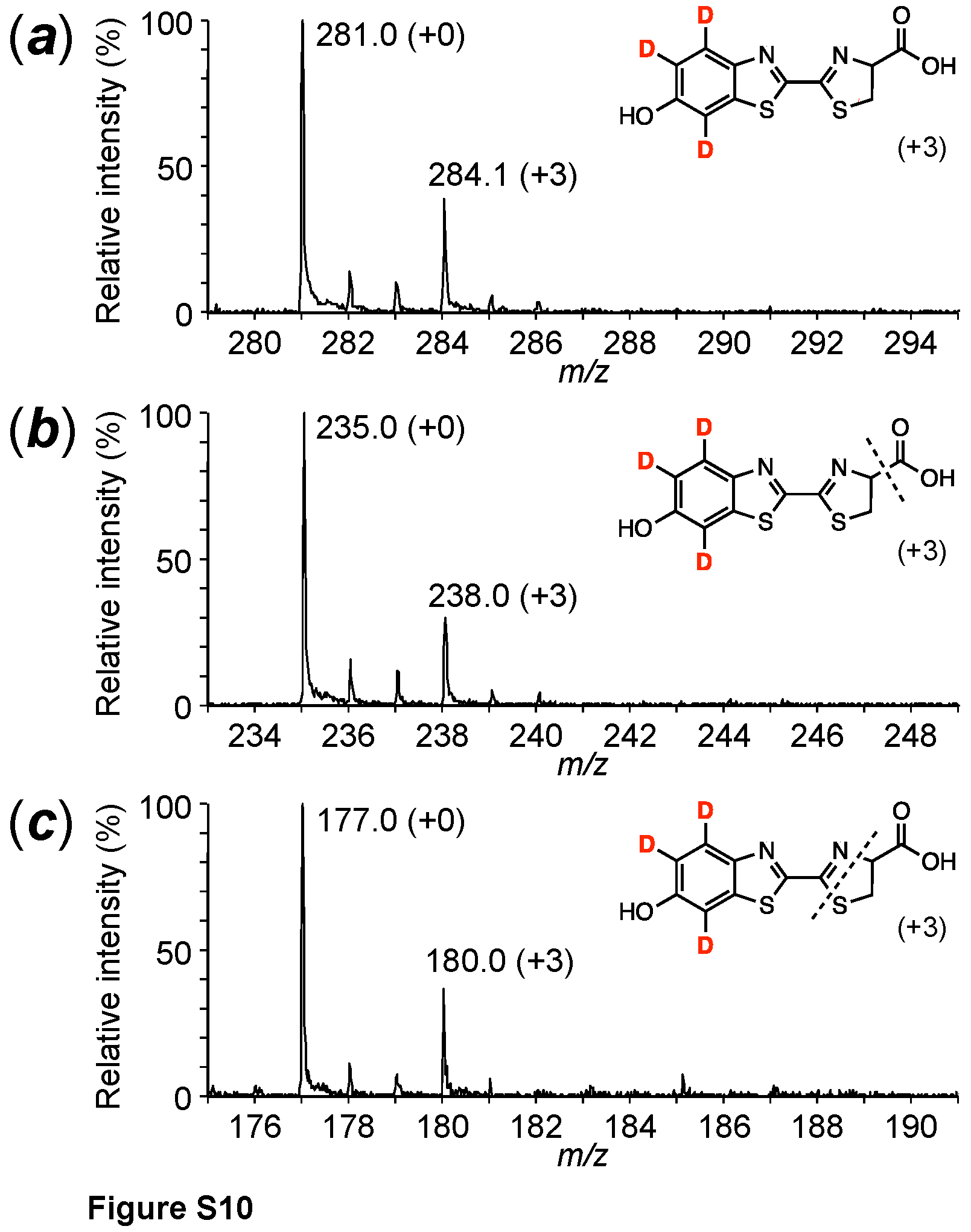

Supplement: Figure S10 — Injecting of p -[D4]-benzoquinone and ʟ-Cys into an adult lantern of L. lateralis. (a), the parent ion of firefly luciferin; (b) and (c), the fragment ions of firefly luciferin. (TIF) [file pone.0084023.s010.tif]

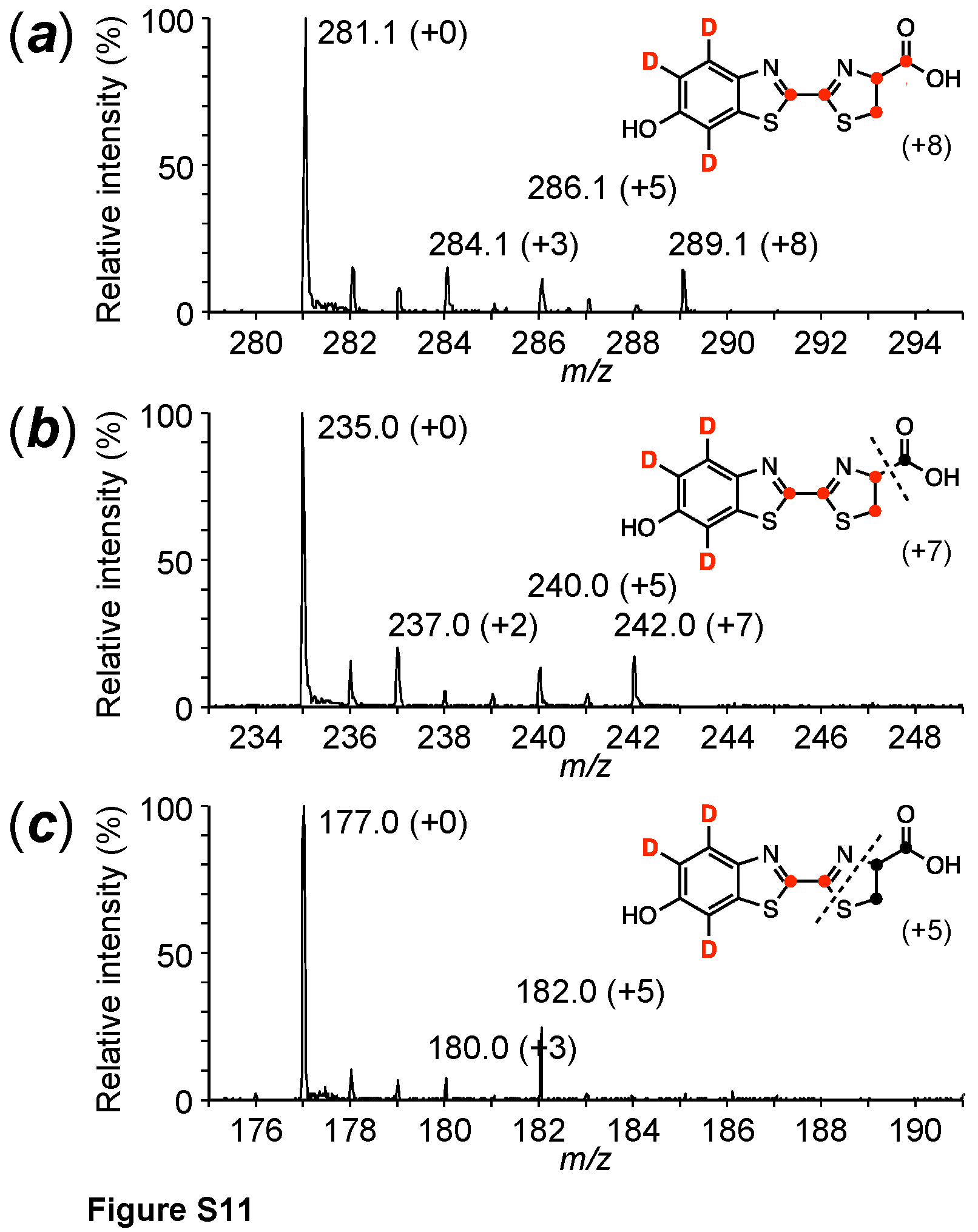

Supplement: Figure S11 — Injecting of 1,4-[D6]-hydroquinone and ʟ-Cys[U-13C3] into an adult lantern of L. lateralis. (a), the parent ion of firefly luciferin; (b) and (c), the fragment ions of firefly luciferin. (TIF) [file pone.0084023.s011.tif]

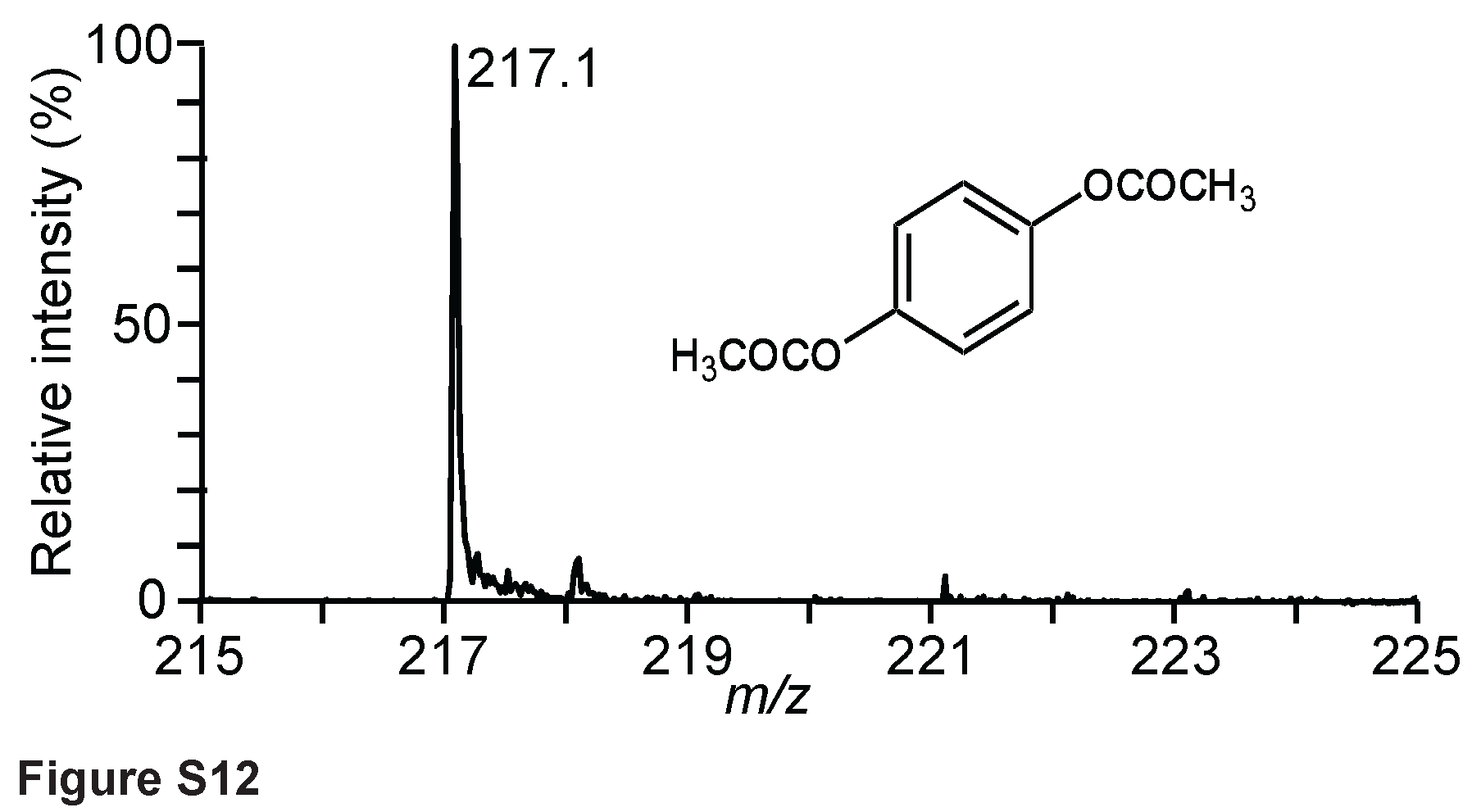

Supplement: Figure S12 — Mass spectrum of acetylated 1,4-hydroquinone isolated from arbutin in L. lateralis . (TIF) [file pone.0084023.s012.tif]

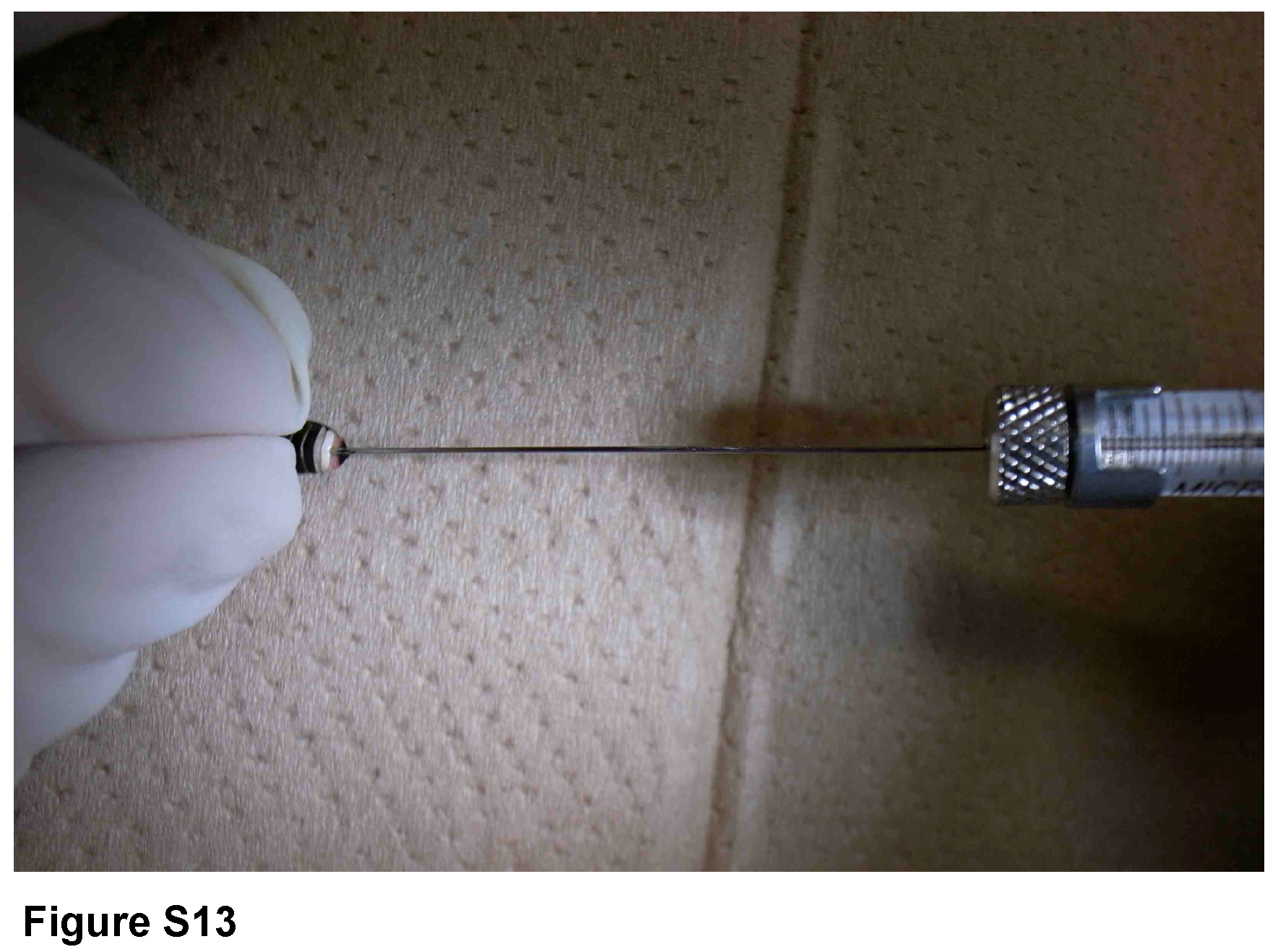

Supplement: Figure S13 — Photograph of the procedure to inject the chemicals into the adult lantern of L. lateralis (female) using a syringe. (TIF) [file pone.0084023.s013.tif]
