# Supplementary material for: Biosynthesis of Firefly Luciferin in Adult Lantern: Decarboxylation of ʟ-Cysteine is a Key Step for Benzothiazole Ring Formation in Firefly Luciferin Synthesis
Source: PLoS One. 2013 Dec 31;8(12):e84023. doi: 10.1371/journal.pone.0084023 (PMC3877152; doi:10.1371/journal.pone.0084023)
Supplement: Table S1 — Relative isotopic peak intensities (%) of calculated firefly luciferin, synthetic ᴅ-firefly luciferin and ʟ-firefly luciferin. (DOC) [file pone.0084023.s014.doc]

**Supplementary Table S1.**

Relative isotopic peak intensities (%) of calculated firefly luciferin, synthetic D-firefly luciferin and L-firefly luciferin.

| Number of stable isotope atom in MH^+^ | Calculated for firefly luciferin ^a^ | | | Authentic D-firefly luciferin ^b^ | | | Authentic L-firefly luciferin ^b^ | | |
| --- | --- | --- | --- | --- | --- | --- | --- | --- | --- |
|  | (***a***) ^c^ | (***b***) ^c^ | (***c***) ^c^ | (***a***) | (***b***) | (***c***) | (***a***) | (***b***) | (***c***) |
| +0 | 100.0 | 100.0 | 100.0 | 100.0 | 100.0 | 100.0 | 100.0 | 100.0 | 100.0 |
| +1 | 14.8 | 13.6 | 10.5 | 14.5 | 17.5 | 14.9 | 18.0 | 17.1 | 18.4 |
| +2 | 10.5 | 9.9 | 5.1 | 11.0 | 13.4 | 11.3 | 11.1 | 9.7 | 5.4 |
| +3 | 1.4 | 1.2 | – | – | 4.2 | – | – | 4.8 | – |
| +4 | – | – | – | – | – | – | – | – | – |

^a^ Relative isotopic peak intensities are calculated based on natural isotopic abundance.

^b^ Chemical synthesized D-firefly luciferin and L-firefly luciferin is used as an authentic sample.

^c^ (***a***) represents the parent mass of firefly luciferin with MH^+^ 281 (+0, 100%). (***b***) and (***c***) represent the fragment ion mass from firefly luciferin with MH^+^ 235 (+0, 100%) and MH^+^ 177 (+0, 100%), respectively.
