# Supplementary material for: Correction: Biosynthesis of Firefly Luciferin in Adult Lantern: Decarboxylation of L-Cysteine is a Key Step for Benzothiazole Ring Formation in Firefly Luciferin Synthesis
Source: PLoS One. 2014 Apr 7;9(4):e95063. doi: 10.1371/journal.pone.0095063 (PMC3978032; doi:10.1371/journal.pone.0095063)
Supplement: File S2 — Republished corrected article. (PDF) [file pone.0095063.s002.pdf]

Biosynthesis of Firefly Luciferin in Adult Lantern: Decarboxylation of L-Cysteine Is a Key Step for Benzothiazole Ring Formation in Firefly Luciferin Synthesis

Yuichi Oba[1]*, Naoki Yoshida[1], Shusei Kanie[1], Makoto Ojika[1], Satoshi Inouye[2]*

1 Graduate School of Bioagricultural Sciences, Nagoya University, Nagoya, Japan, **2** Yokohama Research Center, JNC Corporation, Yokohama, Japan

Abstract

Background: Bioluminescence in fireflies and click beetles is produced by a luciferase-luciferin reaction. The luminescence property and protein structure of firefly luciferase have been investigated, and its cDNA has been used for various assay systems. The chemical structure of firefly luciferin was identified as the D-form in 1963 and studies on the biosynthesis of firefly luciferin began early in the 1970's. Incorporation experiments using ^{14}C-labeled compounds were performed, and cysteine and benzoquinone/hydroquinone were proposed to be biosynthetic component for firefly luciferin. However, there have been no clear conclusions regarding the biosynthetic components of firefly luciferin over 30 years.

Methodology/Principal Findings: Incorporation studies were performed by injecting stable isotope-labeled compounds, including L-[U-$^{13}C_3$]-cysteine, L-[1-^{13}C]-cysteine, L-[3-^{13}C]-cysteine, 1,4-[D_6]-hydroquinone, and *p*-[2,3,5,6-D]-benzoquinone, into the adult lantern of the living Japanese firefly *Luciola lateralis*. After extracting firefly luciferin from the lantern, the incorporation of stable isotope-labeled compounds into firefly luciferin was identified by LC/ESI-TOF-MS. The positions of the stable isotope atoms in firefly luciferin were determined by the mass fragmentation of firefly luciferin.

Conclusions: We demonstrated for the first time that D- and L-firefly luciferins are biosynthesized in the lantern of the adult firefly from two L-cysteine molecules with *p*-benzoquinone/1,4-hydroquinone, accompanied by the decarboxylation of L-cysteine.

Citation: Oba Y, Yoshida N, Kanie S, Ojika M, Inouye S (2013) Biosynthesis of Firefly Luciferin in Adult Lantern: Decarboxylation of L-Cysteine Is a Key Step for Benzothiazole Ring Formation in Firefly Luciferin Synthesis. PLoS ONE 8(12): e84023. doi:10.1371/journal.pone.0084023

Editor: Fanis Missirlis, CINVESTAV-IPN, Mexico

Received October 11, 2013; **Accepted** November 7, 2013; **Published** December 31, 2013

Copyright: © 2013 Oba et al. This is an open-access article distributed under the terms of the Creative Commons Attribution License, which permits unrestricted use, distribution, and reproduction in any medium, provided the original author and source are credited.

Funding: This work was supported in part by a grant from Japan Society for the Promotion of Science (23310159). No additional external funding received for this study. The funders had no role in study design, data collection and analysis, decision to publish, or preparation of the manuscript.

Competing Interests: The authors have the following interests: Satoshi Inouye is employed by JNC Corporation. This does not alter the authors' adherence to all the PLOS ONE policies on sharing data and materials.

* E-mail: sinouye@jnc-corp.co.jp (SI); oba@agr.nagoya-u.ac.jp (YO)

Introduction

Bioluminescence is the emission of visible light produced by living organisms [1,2]. Among insects, the luminous species have been found in three Coleoptera families: Lampyridae (firefly), Elateridae (click beetle), and Phengodidae (railroad worm) [3]. Light emission in these insects is produced by an enzymatic reaction of a luciferase (enzyme) and a luciferin (substrate). The luminescence system is essentially the same with an identical luciferin, ATP, Mg^{2+}, and a highly conserved luciferase [4]. The luciferin is referred to as "firefly luciferin" or "beetle luciferin", and the chemical structure has been identified as (*S*)-2-(6′-hydroxy-2′-benzothiazolyl)-2-thiazoline-4-carboxylic acid (**I**, D-firefly luciferin), which consists of two structural units, benzothiazole and thiazoline rings (Figure 1A). The chirality of the carboxyl group in natural firefly luciferin was determined to be the *S* form by the chemical synthesis of D-firefly luciferin from 2-cyano-6-hydroxybenzothiazole (**III**) and D-cysteine [5,6]. L-Firefly luciferin with the *R* form is not used for the luminescence reaction by firefly luciferase [7]. Thus, firefly luciferase oxidizes only D-firefly luciferin to emit light and produces oxyluciferin (**II**) and CO_2 (Figure 1A).

On the other hand, in marine luminous organisms, Cypridina luciferin and coelenterazine are widely used in the luciferase reactions [2]. Coelenterazine is also used as the light-emitting substrate for the Ca^{2+}-binding photoproteins such as aequorin [8]. Recently, we have been studying the biosyntheses of Cypridina luciferin and coelenterazine in living specimens by feeding experiments using stable isotope-labeled compounds. The incorporation of stable isotopes into the luciferin was determined by mass spectrometry [9–12]. In the luminous ostracods *Cypridina* (presently *Vargula*) *hilgendorfii* and *Cypridina noctiluca*, we concluded that Cypridina luciferin is biosynthesized from the natural amino acids of L-tryptophan, L-arginine, and L-isoleucine [9–11,13]. Further, we demonstrated that coelenterazine is biosynthesized from two L-tyrosines and L-phenylalanine in the deep-sea luminous copepod *Metridia pacifica* [12]. Thus, similar to the method using the radioisotope-labeled compounds, the method of mass spectral analysis accompanied by the incorporation of stable isotope-

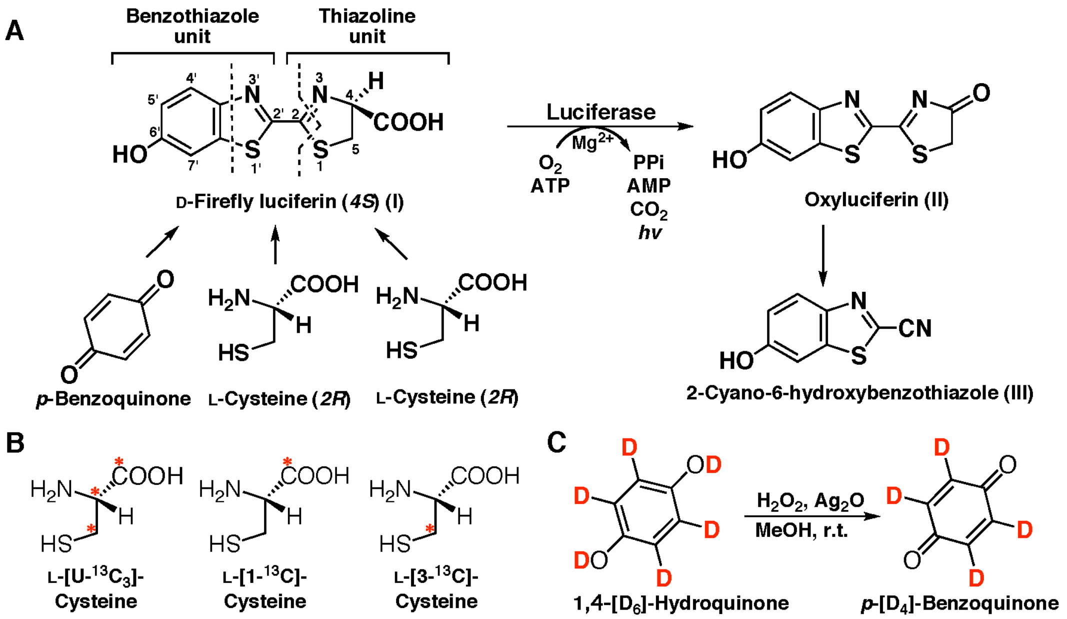

Figure 1. Strategy to study on the biosynthetic pathway of firefly luciferin in an adult lantern of a living firefly by injecting the stable isotope-labeled compounds, and the bioluminescence reaction catalyzed by firefly/beetle luciferase. A. Proposed biosynthetic pathway of firefly luciferin (**I**) from *p*-benzoquinone and two L-cysteines in an adult lantern, and the luminescence reaction of luciferin with firefly luciferase, followed by the formation of 2-cyano-6-hydroxybenzothiazole (**III**) from oxyluciferin (**II**). B. Stable isotope-labeled L-cysteines used in the experiments. Asterisk indicates the position of a ^{13}C atom. C. Preparation of *p*-[D_4]-benzoquinone from 1,4-[D_6]-hydroquinone by the oxidation reaction using silver oxide with H_2O_2.
doi:10.1371/journal.pone.0084023.g001

labeled compounds is useful for investigating the biosynthetic process. With regard to studies on the biosynthesis of luciferin in firefly and click beetle, four biochemical investigations involving incorporation experiments with ^{14}C-labeled compounds have been reported [14–17] and studies using biomimetic synthesis have also been reported [16,18].

The initial study on the biosynthesis of firefly luciferin was reported in 1974 [14]. Based on the chemical synthesis of D-firefly luciferin from 2-cyano-6-hydroxybenzothiazole and D-cysteine [6], [2-^{14}C]-oxyluciferin and 2-[cyano-^{14}C]-6-hydroxybenzothiazole were chemically synthesized and were injected into the adult lantern of the Japanese firefly *Luciola cruciata*. To determine the incorporation of ^{14}C-labeled compounds into firefly luciferin, ^{14}C-labeled firefly luciferin with an excess of cold D-firefly luciferin was converted to its diacetate derivative and crystallized, following which the radioactivity was determined [14]. From these results, 2-cyano-6-hydroxybenzothiazole (**III**) was proposed to be a candidate for the biosynthetic precursor of firefly luciferin, and oxyluciferin (**II**) could be regenerated to luciferin through 2-cyano-6-hydroxybenzothiazole in the firefly lantern. Furthermore, when cell-free extracts from the frozen lanterns were incubated with ^{14}C-oxyluciferin and cysteine in the presence of ATP, the incorporation of ^{14}C-oxyluciferin into firefly luciferin was increased. However, the following controversial points exist in this report: (i) The configuration of L- or D-luciferin has not been not mentioned in the report. (ii) oxyluciferin is degraded to 2-cyano-6-hydroxybenzothiazole (**III**) under non-enzymatic conditions at pH 7–9. (iii) the condensation of 2-cyano-6-hydroxybenzothiazole (**III**) with D- and L-cysteine proceeds spontaneously in aqueous solutions (pH 8) at room temperature [6,14] and forms D- and L-firefly luciferin, respectively; and (iv) the presence of 2-cyano-6-hydroxybenzothiazole (**III**) or an intermediate of its derivatives has not been identified in the firefly. Thus, it is still unclear whether oxyluciferin and 2-cyano-6-hydorxybenzothiazole (**III**) are not intermediates for luciferin biosynthesis and recycling intermediates from oxyluciferin to luciferin in a living firefly [19,20].

In the second report [15], the injection experiments of *p*-[2,3,5,6-^{14}C]-benzoquinone, 1,4-[2,3,5,6-^{14}C]-hydroquinone, L-[U-$^{14}C_6$]-tyrosine, and sodium [2-^{14}C]-acetate into adult specimens of *L. cruciata* were performed. The results indicated that *p*-benzoquinone and 1,4-hydroquinone are candidates for the biosynthetic component of firefly luciferin [15]. However, there are no descriptions about the configuration of cysteine and its incorporation into the benzothiazole ring in this report [15]. In addition, the presence of *p*-benzoquinone or 1,4-hydroquinone in the firefly has not been reported.

For studies on the biosynthesis of beetle luciferin, the luminous click beetle *Pyrophorus pellucens* was used in 1976 [16]. The biosynthesis of luciferin was examined by feeding experiments using the adult specimen of *P. pellucens* with a 10% sucrose solution containing D/L-[1-^{14}C]-cystine (a dimer of D- and/or L-cysteine). After the addition of an excess of D-luciferin into the extracts of photophores, the ^{14}C-labeled luciferin recovered by TLC was crystallized and the radioactivity was determined. The results suggested that D/L-cystine was reduced to D- and L-cysteine and they were incorporated into beetle luciferin and that D- and/or L-cysteine are a biosynthetic unit of luciferin. In this report, the important point was that the possibility of the decarboxylation from a cysteine was predicted during benzothiazole ring formation. However, the configuration of cysteine incorporated in beetle luciferin and the incorporation of cysteine into the benzothiazole ring with decarboxylation were not revealed. Further, in 1988, the injection experiment of [U-$^{14}C_6$]-cystine into the larvae of the luminous click beetle *Pyrearinus termitilluminans* was performed, and the ^{14}C-labeled luciferin was extracted and was determined by

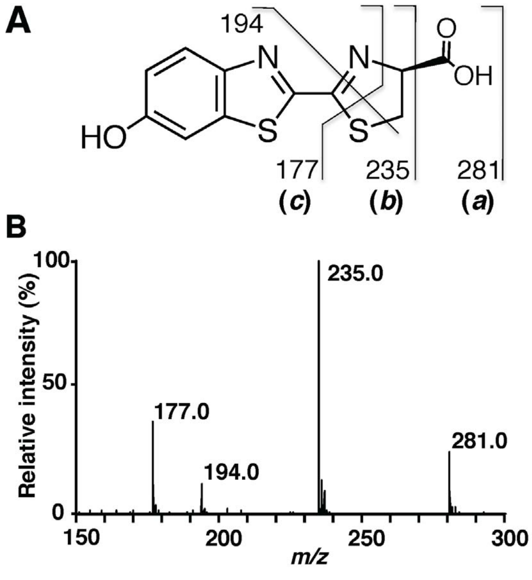

Figure 2. Mass spectrum of D-firefly luciferin by ESI-TOF-MS analysis. A. The structure of D-firefly luciferin and the predicted mass fragment ions. The parent ion of D-luciferin at *m/z* 281 $(M+H)^+$ (***a***), and its fragment ions at *m/z* 235 (***b***) and 177 (***c***). B. ESI-TOF-MS analysis of synthetic D-firefly luciferin.
doi:10.1371/journal.pone.0084023.g002

TLC without the addition of luciferin [17]. As a result, cysteine from [U-$^{14}C_6$]-cystine was incorporated into luciferin, similar to the case of adult *P. pellucens* [16]. Unfortunately, the configuration of [U-$^{14}C_6$]-cystine used was not described in the report and the number of [U-$^{14}C_3$]-cysteine incorporated into luciferin was not determined.

In this study, we incorporated stable isotope-labeled L-cysteine, *p*-benzoquinone, and 1,4-hydroquinone into firefly luciferin in the adult lantern of a living firefly, and identified the positions of the stable isotopes incorporated into firefly luciferin by LC/ESI-TOF-MS analysis. We revealed that D- and L-firefly luciferins (beetle luciferin) are biosynthesized from *p*-benzoquinone/1,4-hydorquinone with two L-cysteines, accompanied by the decarboxylation of L-cysteine.

Results

Injection of Stable Isotope-labeled Compounds into an Adult Lantern of *L. lateralis*

For incorporation experiments into firefly luciferin in an adult lantern of the firefly, the stable isotope-labeled compounds of L-Cys[U-$^{13}C_3$], L-Cys[1-^{13}C], L-Cys[3-^{13}C] and/or [D_6]-hydroquinone were used (Figure 1B and 1C). [D_4]-Benzoquinone was chemically synthesized from [D_6]-hydroquinone by oxidation with H_2O_2 and Ag_2O (Figure 1C). One microliter of each isotopic compounds dissolved in sterile H_2O was injected into the body cavity of an adult lantern using a syringe. The injected specimens were kept in a moisture chamber for 24 h, and the survived specimen was used for mass spectral analysis. The amount of the compounds injected was 550 nmol per specimen, except for *p*-benzoquinone which was 55 nmol. Owing to the toxicity of *p*-benzoquinone, the specimens injected with 550 nmol of *p*-benzoquinone were hardly survived for 24 h.

LC/ESI-TOF-MS Analysis of Stable Isotope-labeled Firefly Luciferins in the Lantern

We have reported that a single specimen of the adult *L. lateralis* contains approximately 0.5 nmol of firefly luciferin [21] and this amount is enough for analysis by LC/ESI-TOF-MS under our experimental conditions. Chemically synthesized D- and L-firefly luciferins were used as authentic samples to obtain the standard mass spectrum by LC/ESI-TOF-MS (Figure 2 and Table S1 and Figure S1–S4). As shown in Fig. 2, the parent ion of D-firefly luciferin was observed at *m/z* 281 ((***a***) in Figure 2A). The fragment ions were formed at *m/z* 235 ((***b***) in Figure 2A), *m/z* 194 and *m/z* 177 ((***c***) in Figure 2A) by increasing the voltage of nozzle potential up to 360 V. The isotopic fragment ions of (***b***) and (***c***) were used for determining the positions of the ^{13}C-labeled atom in firefly luciferin. In our injection experiments, the incorporation efficiencies of stable isotope-labeled compounds into luciferin were estimated to be between 7% and 48% by calculating the peak intensities of the isotopic ions. The incorporation experiments were repeated 2–3 times to confirm reproducibility.

Incorporation of L-Cys[U-$^{13}C_3$] into Firefly Luciferin in the Presence of Non-isotopic 1,4-hydroquinone or p-benzoquinone

To determine whether L-cysteine is a biosynthetic component for both 6-hydroxybenzothiazole and 2-thiazoline-4-carboxylate moieties in firefly luciferin (Figure 1A), the incorporation experiments were performed with L-Cys[U-$^{13}C_3$] in the presence and absence of 1,4-hydroquinone or *p*-benzoquinone. Because *p*-benzoquinone shows high toxicity in living organisms, the concentration of *p*-benzoquinone injected was 10-fold lower than that of 1,4-hydroquinone. The results of ESI-TOF-MS analysis are summarized in Table 1.

(i) Injection of L-Cys[U-$^{13}C_3$]: The intensities of the isotopic parent ion (***a***) at *m/z* 284 (+3, 8.4%) and the isotopic fragment ion (***b***) at *m/z* 237 (+2, 15.0%) were mainly increased; however, no significant increase in the isotopic fragment ion (***c***) was observed. These results indicated that one cysteine molecule was incorporated into the 2-thiazoline-4-carboxylate moiety but not the 6-hydroxybenzothiazole moiety in firefly luciferin (Table 1 & Figure S5). This result also indicated that firefly luciferin was generated from an unidentified compound having a benzothiazole and L-cysteine in the lantern.

(ii) Injection of L-Cys[U-$^{13}C_3$] and 1,4-hydroquinone: The addition of non-isotopic 1,4-hydroquinone stimulated the incorporation efficiency of L-Cys[U-$^{13}C_3$] into firefly luciferin (Figure 3A). The isotopic parent ions (***a***) at *m/z* 283 (+2, 33.3%), 284 (+3, 32.0%) and 286 (+5, 38.0%) indicated that two L-cysteine molecules were incorporated into firefly luciferin. The fragment ions (***b***) at *m/z* 237 (+2, 62.4%) and 239 (+4, 31.8%) and (***c***) at *m/z* 179 (+2, 51.5%) indicated that one carbon atom was eliminated from one of the two L-cysteine molecules during the incorporation into firefly luciferin (Figure 4). Thus, L-cysteine was independently incorporated into 6-hydroxybenzothiazole and 2-thiazoline-4-carboxylate moieties.

(iii) Injection of L-Cys[U-$^{13}C_3$] and *p*-benzoquinone: The incorporation pattern and efficiency of L-Cys[U-$^{13}C_3$] and *p*-benzoquinone into firefly luciferin were similar to those of L-Cys[U-$^{13}C_3$] and 1,4-hydroquinone, indicating that *p*-benzoquinone is also a biosynthetic component in firefly luciferin (Figure S6). It is known that 1,4-hydroquinone is

Table 1. Relative isotopic peak intensities (%) of the parent and its fragment mass from firefly luciferin in the lantern extracts after injecting L-Cys[U-$^{13}C_3$] with 1,4-hydroquinone or *p*-benzoquinone into the adult of *L. lateralis*.

Number of stable isotope atom in MH^+	Without injection			L-Cys[U-$^{13}C_3$]			L-Cys[U-$^{13}C_3$] +1,4-hydroquinone			L-Cys[U-$^{13}C_3$]+*p*-benzoquinone		
	(*a*) [a]	(*b*) [a]	(*c*) [a]	(*a*)	(*b*)	(*c*)	(*a*)	(*b*)	(*c*)	(*a*)	(*b*)	(*c*)
+0	100.0	100.0	100.0	100.0	100.0	100.0	100.0	100.0	100.0	100.0	100.0	100.0
+1	14.9	15.1	9.5	15.9	16.7	9.4	14.4	11.1	10.1	15.1	13.5	12.5
+2	9.8	9.6	5.1	9.7	**15.0**	4.1	**33.3**	**62.4**	**51.5**	**42.4**	**58.4**	**63.1**
+3	1.6	1.3	–	**8.4**	1.8	–	**32.0**	6.1	5.4	**20.1**	11.4	5.1
+4	–	–	–	2.5	–	–	8.9	**31.8**	–	8.8	**41.3**	–
+5	–	–	–	–	–	–	**38.0**	3.7	–	**39.3**	5.5	–
+6	–	–	–	–	–	–	7.6	3.3	–	5.9	2.8	–
+7	–	–	–	–	–	–	3.1	–	–	4.3	–	–
+8	–	–	–	–	–	–	–	–	–	–	–	–

[a](*a*) represents the parent mass of firefly luciferin with MH^+281 (+0, 100%). (*b*) and (*c*) represent the fragment ion mass from firefly luciferin with MH^+235 (+0, 100%) and MH^+177 (+0, 100%), respectively, as shown in Fig. 2. The numbers in bold are the significant mass peaks containing the incorporated stable isotope atoms.
doi:10.1371/journal.pone.0084023.t001

enzymatically oxidized to produce *p*-benzoquinone in living cells. The increase in the incorporation efficiency of L-Cys[U-$^{13}C_3$] into firefly luciferin by the addition of 1,4-hydroquinone or *p*-benzoquinone showed that they are other biosynthetic components of firefly luciferin.

Incorporation of [D_6]-hydroquinone or [D_4]-benzoquinone into Firefly Luciferin in the Presence of Non-isotopic L-cysteine (L-Cys)

To confirm 1,4-hydroquinone and *p*-benzoquinone as biosynthetic components for firefly luciferin, the injection experiments of [D_6]-hydroquinone and [D_4]-benzoquinone with L-cysteine were performed as follows (Table 2).

(i) Injection of [D_6]-hydroquinone: The isotopic parent ion (***a***) at *m/z* 284 (+3, 8.8%) and the fragment ions (***b*** and ***c***) at *m/z* 238 (+3, 8.4%) and 180 (+3, 7.3%), respectively, indicated that [D_6]-hydroquinone was incorporated into firefly luciferin (Figure 5B). The luciferin was biosynthesized from [D_6]-hydroquinone and endogenous cysteine in the adult lantern (Figure S7).

(ii) Injection of [D_6]-hydroquinone and L-Cys: The addition of non-isotopic L-cysteine stimulated the incorporation of [D_6]-hydroquinone into firefly luciferin (Figure 5B), similar to the case of the addition of 1,4-hydroquinone with L-Cys[U-$^{13}C_3$] (Figure 3A and Figure S8).

(iii) Injection of [D_4]-benzoquinone: The isotopic parent ion (***a***) at *m/z* 284 (+3, 22.0%) and the fragment ions (***b*** and ***c***) at *m/z* 238 (+3, 24.2%) and 180 (+3, 27.1%), respectively, indicated that [D_4]-benzoquinone was incorporated into firefly luciferin (Figure 5A), similar to from the case of [D_6]-hydroquinone (Figure S9). Thus, *p*-benzoquinone is also a biosynthetic component for firefly luciferin.

(iv) Injection of [D_4]-benzoquinone and L-Cys: The isotopic ion patterns of ***a***, ***b*** and ***c*** from labeled firefly luciferin were similar to those in the injection experiments using [D_6]-hydroquinone, [D_6]-hydroquinone with L-Cys, and [D_4]-benzoquinone (Figure S10).

Notably, the incorporation efficiency of [D_4]-benzoquinone into firefly luciferin (Figure S9) was higher than that of [D_6]-hydroquinone (Table 2, Figure S7), despite the fact that the amount of *p*-benzoquinone injected was 10 times lower than that of 1,4-hydroquinone. This result indicated that *p*-benzoquinone might be preferred over 1,4-hydroquinone for firefly luciferin synthesis, and that 1,4-hydroquinone may converted to *p*-benzoquinone and immediately used for the biosynthesis of firefly luciferin in the lantern.

Incorporation of L-Cys[1-^{13}C] or L-Cys[3-^{13}C] into Firefly Luciferin

It has been proposed that one carbon atom is eliminated from cysteine during the biosynthesis of firefly luciferin [16]. To identify the carbon atom eliminated from L-cysteine through 6-hydroxybenzothiazole formation, the incorporation studies with L-Cys[1-^{13}C] and L-Cys[3-^{13}C] were performed in the presence of 1,4-hydroquinone (Figure 3B, C and Table 3). When L-Cys[1-^{13}C] and 1,4-hydroquinone were injected, the isotopic parent ion (***a***) at *m/z* 282 (+1, 68.6%) was increased; however, the fragment ions (***b*** and ***c***) at *m/z* 236 (+1, 16.3%) and 178 (+1, 10.9%), respectively, were not increased (Table 3 and Figure 6).

In contrast, the injection of L-Cys[3-^{13}C] resulted in mass increases of the isotopic parent ions at *m/z* 282 (+1, 63.2%) and 283 (+2, 42.7%) and the fragment ions at *m/z* 236 (+1, 60.6%), 237 (+2, 43.3%) and 178 (+1, 42.2%) (Table 3 and Figure 7). These results indicated that the carboxyl group of L-cysteine was eliminated during the formation of the benzothiazole ring in firefly.

Incorporation of [D_6]-hydroquinone and L-Cys[U-$^{13}C_3$] into Firefly Luciferin

A double-labeling experiment using [D_6]-hydroquinone and L-Cys[U-$^{13}C_3$] was performed to confirm the *de novo* synthesis of firefly luciferin in the firefly lantern (Figure 5C and Table 2). The peak intensities of the isotopic parent ions (***a***) at *m/z* 284 (+3, 15.5%), 286 (+5, 11.7%) and 289 (+8, 14.4%), the fragment ions (***b***) at *m/z* 237 (+2, 20.0%), 240 (+5, 13.4%) and 242 (+7, 17.2%), and the fragment ions (***c***) at *m/z* 180 (+3, 7.4%) and 182 (+5, 24.5%) were increased, indicating that one hydroquinone and two

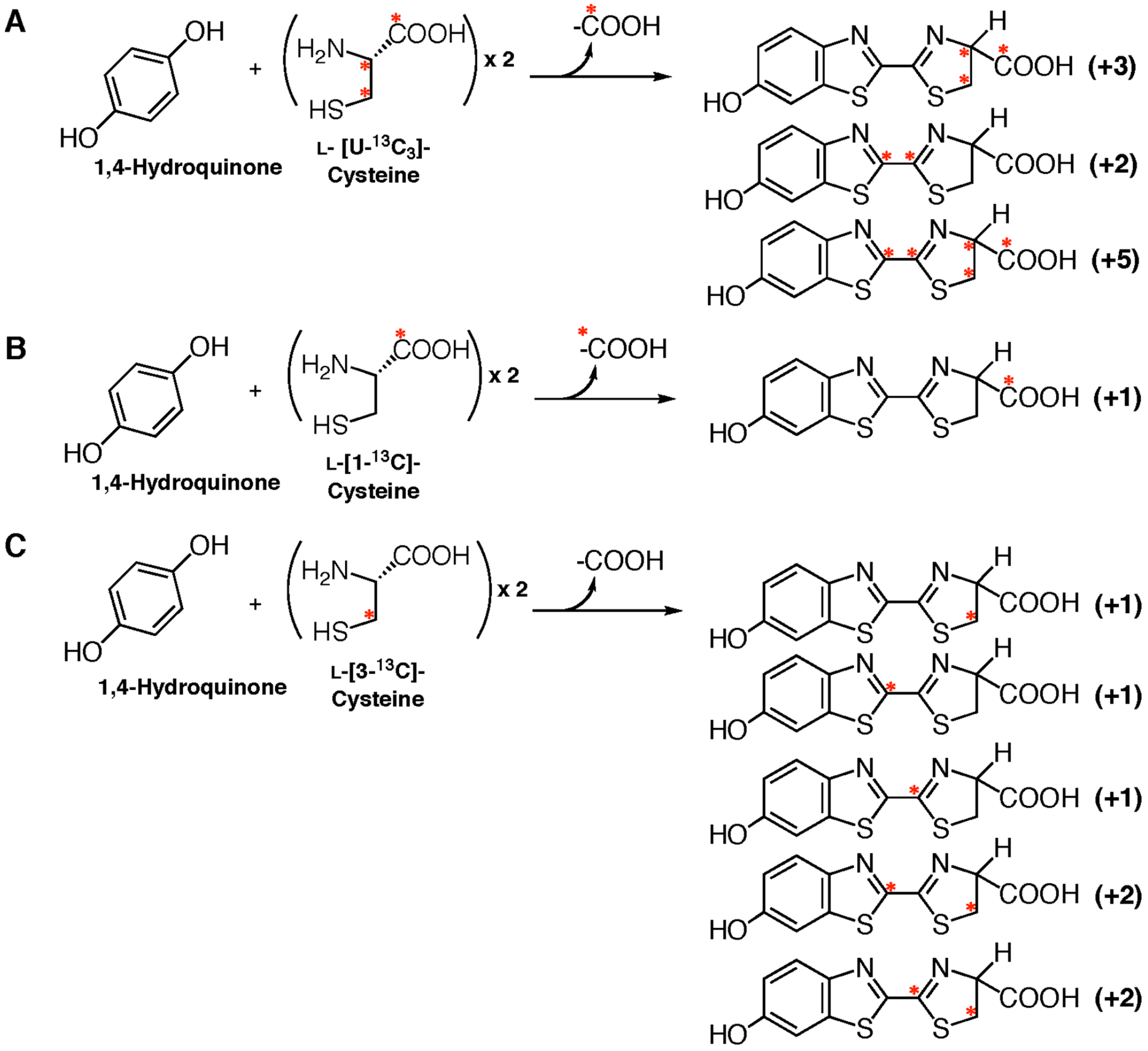

Figure 3. Incorporation of 1,4-hydroquinone and ^{13}C-labeled L-cysteines into firefly luciferin in an adult lantern of *L. lateralis*. A. Predicted firefly luciferins incorporated from 1,4-hydroquinone and L-Cys[U-$^{13}C_3$]. B. Predicted firefly luciferins incorporated from 1,4-hydroquinone and L-Cys[1-^{13}C]. C. Predicted firefly luciferins incorporated from 1,4-hydroquinone and L-Cys[3-^{13}C]. The number in parenthesis on the right indicates the number of ^{13}C-atom incorporated into firefly luciferin. Asterisk indicates the position of a ^{13}C-atom.
doi:10.1371/journal.pone.0084023.g003

L-cysteine molecules were incorporated into firefly luciferin (Figure 8 and Figure S11). Thus, two L-cysteine molecules and 1,4-hydroquinone are required for the *de novo* synthesis of firefly luciferin.

Identification of Endogenous D- and L-firefly Luciferin in an Adult Lantern in *L. lateralis* and Incorporation of L-Cys[U-$^{13}C_3$] into D- and L-firefly Luciferin

To characterize the chirality of firefly luciferin, firefly luciferin was extracted from the adult lantern of *L. lateralis* without racemization between D- and L-luciferin (see experimental section), following which then D- and L-luciferins were separated by HPLC with a chiral column (Figure 9A). The peak ratio of D-luciferin to L-luciferin was approximately 9:1 (Figure 9A–c), indicating that L-luciferin was present in an adult lantern. Following this, an incorporation study of L-Cys[U-$^{13}C_3$] and 1,4-hydroquinone was performed and the peak ratio of D-luciferin to L-luciferin was changed to 7:3 with an increase in L-luciferin (Figure 9A–d). These peak fractions were collected and subsequently subjected to LC/ESI-TOF-MS analysis (Figure 9B). Interestingly, L-cysteine was incorporated into not only L-luciferin but also D-luciferin, indicating that L-cysteine is a biosynthetic component of D-luciferin.

Identification of Free 1,4-hydroquinone and Arbutin in Firefly Lantern

As described above, 1,4-hydroquinone is a biosynthetic component of firefly luciferin. To examine the presence of free 1,4-hydroquinone or its storage forms such as arbutin in the lantern of an adult firefly, we analyzed the lantern extracts by HPLC. Under our analytical conditions, free 1,4-hydroquinone was not detected in the lantern extracts. However, we successfully detected arbutin in the extracts by HPLC analysis (Figure 10A). After the arbutin fraction was hydrolyzed with HCl (Figure 10C), the hydrolyzed sample was subjected to

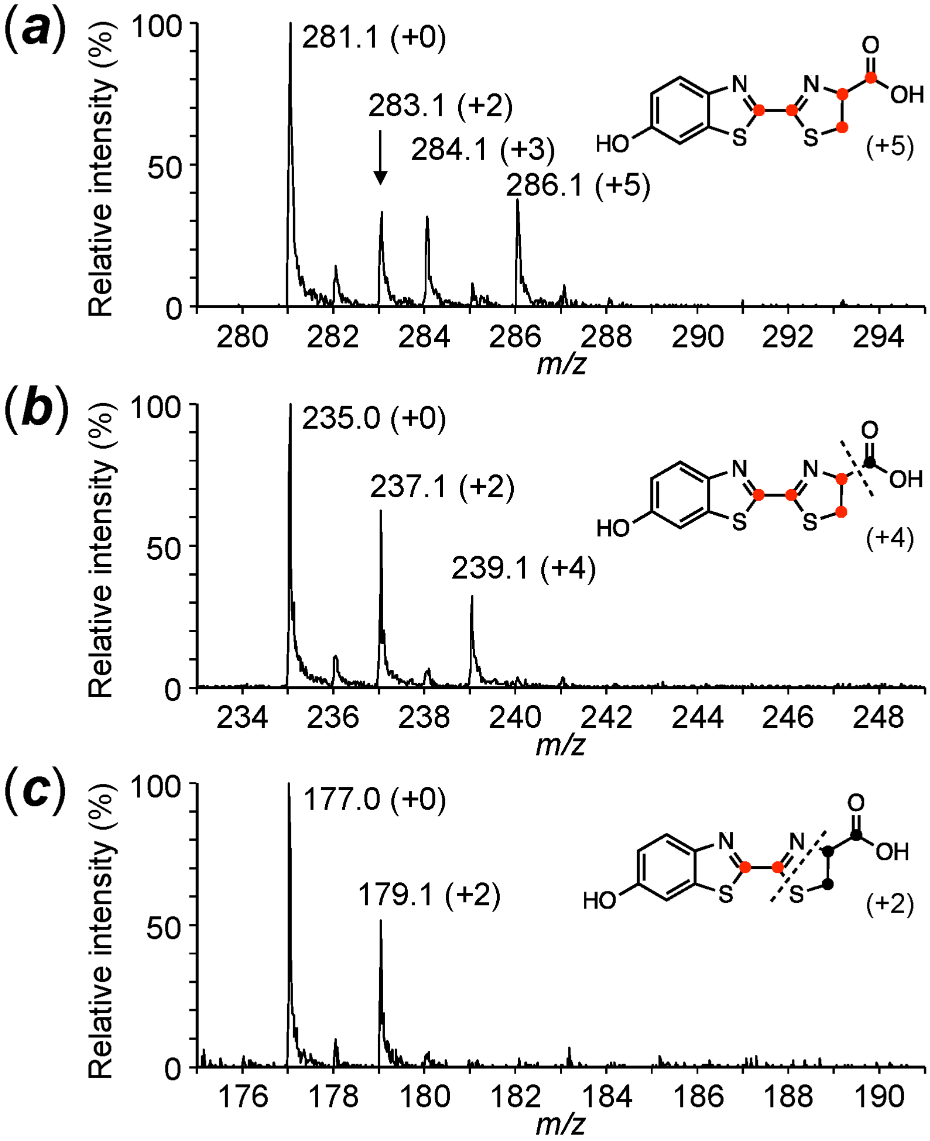

Figure 4. Mass spectra of firefly luciferin labeled with L-Cys[U-$^{13}C_3$] and 1,4-hydroquinone in the adult lantern of *L. lateralis*. (*a*), the parent ion; (*b*) and (*c*), the fragments ions of firefly luciferin.
doi:10.1371/journal.pone.0084023.g004

HPLC analysis and the fluorescence peak of 1,4-hydroquinone was detected (Figure 10B). Furthermore, the structure of 1,4-hydroquinone in the hydrolyzed sample was confirmed as an acetylated derivative by LC/ESI-TOF-MS (Figure S12). The content of 1,4-hydroquinone after hydrolysis was estimated to be 144±34 pmol per specimen using the standard curve of 1,4-hydroquinone (data not shown). This result suggested that 1,4-hydroquinone would be released from a glycoside derivative such as arbutin in the lantern and used for the biosynthesis of firefly luciferin.

Discussion

Studies on the biosynthesis of luciferin in firefly and click beetle were initiated in the early 1970's using ^{14}C-labeled compounds. A hypothesis that firefly luciferin (beetle luciferin) is biosynthesized from *p*-benzoquinone and two cysteines was proposed [14–16,18]. In this report, we have identified the biosynthetic components of firefly luciferin by mass spectroscopy with stable isotope-labeled compounds. The ^{13}C-labeled L-cysteine, *p*-[D_4]-benzoquinone and 1,4-[D_6]-hydroquinone were incorporated into firefly luciferin in an adult lantern of a firefly. The incorporation experiment with L-Cys[U-$^{13}C_3$] indicated that L-cysteine was incorporated into both the benzothiazole and thiazoline unit of firefly luciferin (Figures 1 and 3). This is the first demonstration that two L-cysteine molecules are the biosynthetic components of firefly luciferin (Figure 3 and Table 2). Furthermore, the incorporation of L-[1-^{13}C]-cysteine and L-[3-^{13}C]-cysteine into firefly luciferin revealed that the carboxyl group of L-[1-^{13}C]-cysteine was eliminated during the benzothiazole ring formation of firefly luciferin (Figure 3 and Table 1), followed by the thiazoline ring formation of firefly luciferin (Figure 11). This result clearly explains the previous observation that the radioisotope activity of ^{14}C-labeled firefly luciferin was lost following acetylation at the carboxyl group of luciferin [16]. Previously, a biosynthetic pathway of firefly

Table 2. Relative isotopic peak intensities (%) of the parent and its fragment mass from firefly luciferin in the lantern extracts after injecting [D_6]-hydroquinone or [D_4]-benzoquinone into the adult of *L. lateralis*.

**Number of stable isotope atom in MH^+**	**[D_6]-hydroquinone**			**[D_6]-hydroquinone + L-Cys**			**[D_6]-hydroquinone + L-Cys[U-$^{13}C_3$]**			**[D_4]-benzoquinone**			**[D_4]-benzoquinone + L-Cys**		
	(*a*)[a]	(*b*)[a]	(*c*)[a]	(*a*)	(*b*)	(*c*)	(*a*)	(*b*)	(*c*)	(*a*)	(*b*)	(*c*)	(*a*)	(*b*)	(*c*)
+0	100.0	100.0	100.0	100.0	100.0	100.0	100.0	100.0	100.0	100.0	100.0	100.0	100.0	100.0	100.0
+1	16.6	15.0	9.1	16.4	14.1	9.3	15.2	15.7	10.5	13.8	13.6	10.5	14.3	15.5	10.7
+2	9.8	9.4	6.3	12.3	13.8	7.8	8.8	**20.0**	6.3	11.3	11.5	4.7	10.3	11.8	6.9
+3	**8.8**	**8.4**	**7.3**	**55.8**	**66.0**	**56.8**	**15.5**	5.0	**7.4**	**22.0**	**24.2**	**27.1**	**38.9**	**29.7**	**36.7**
+4	–	–	–	7.8	8.5	4.7	3.3	3.9	1.1	3.6	3.1	2.6	5.9	4.6	5.8
+5	–	–	–	4.9	6.0	4.8	**11.7**	**13.4**	**24.5**	2.6	2.9	–	3.3	4.1	–
+6	–	–	–	–	–	–	4.6	4.3	2.1	–	–	–	–	–	–
+7	–	–	–	–	–	–	3.0	**17.2**	–	–	–	–	–	–	–
+8	–	–	–	–	–	–	**14.4**	–	–	–	–	–	–	–	–
+9	–	–	–	–	–	–	1.5	–	–	–	–	–	–	–	–
+10	–	–	–	–	–	–	–	–	–	–	–	–	–	–	–

[a](*a*) represents the parent mass of firefly luciferin with MH^+281 (+0, 100%). (*b*) and (*c*) represent the fragment ions mass from firefly luciferin with MH^+235 (+0, 100%) and MH^+177 (+0, 100%), respectively, as shown in Fig. 2. The numbers in bold are the significant mass peaks containing the incorporated stable isotope atoms.
doi:10.1371/journal.pone.0084023.t002

luciferin from *p*-benzoquinone and a dipeptide of cysteine was proposed [18]. This possibility was not acceptable from the evidence that the carboxyl group from L-cysteine was eliminated. However, it is unclear whether the carbon atoms at the C2' in the benzothiazole unit and the C2 in the thiazoline unit were derived from the carbon atom at the C2 or C3 position of L-cysteine in our experiments (Figure 7).

On the other hand, the results of incorporation studies with *p*-[D_4]-benzoquinone and 1,4-[D_6]-hydroquinone were in good agreement with those of a study with ^{14}C-lableled *p*-benzoquinone and 1,4-hydroquinone [15]. Thus, *p*-benzoquinone and 1,4-hydroquinone are components of the benzothiazole unit of firefly luciferin.

It is known that quinones including *p*-benzoquinone and its derivatives are found in some beetles (Coleoptera) [22–24] and *p*-benzoquinone is a metabolite produced by the oxidation of 1,4-hydroquinone [25]. Because *p*-benzoquinone shows high toxicity to living organisms, the concentration of *p*-benzoquinone was 10-fold lower than that of 1,4-hydroquinone in our injection experiments. The incorporation efficiency of *p*-benzoquinone into firefly luciferin was higher than that of 1,4-hydroquinone, suggesting that *p*-benzoquinone may be a preferred substance for the biosynthesis of firefly luciferin in the adult lantern. We detect arbutin, but not 1,4-hydroquionone in the firefly. It is considered that 1,4-hydroquione with low toxicity is stored as a non-toxic form of glycoside such as arbutin and released by the digestive enzyme β-glucosidase [25] and possibly oxidized to *p*-benzoquinone immediately for luciferin synthesis. However, the possibility that 1,4-hydroquinone is a direct biosynthetic precursor still remains.

Recently, the conversion of L-luciferin to D-luciferin in firefly has been proposed by the racemization through L-luciferyl CoA to D-luciferyl CoA, followed by its hydrolysis with an esterase [20]. L-Luciferyl CoA was produced from L-luciferin by "firefly luciferase" in the presence of ATP, Mg^{2+} and CoA, although L-luciferin is a potent inhibitor of firefly luciferase [26,27]. However, another possibility that the D-configuration in luciferin is formed during the thiazoline ring formation accompanied by the conversion of L-form to D-form of cysteine still remains.

In this report, we determined the absolute configuration of the isotope-labeled firefly luciferin by HPLC analysis with a chiral column (Figure 9) and found that L-cysteine was incorporated into not only L-luciferin but also D-luciferin, indicating that L-cysteine is a biosynthetic component of D-luciferin. The mechanism by which D-firefly luciferin is biosynthesized from L-cysteine remains unclear.

In conclusion, we have demonstrated that the 6-hydroxybenzothiazole moiety in D- and L-firefly luciferins is biosynthesized from 1,4-hydroquinone/benzoquinone with L-cysteine, accompanied by the elimination of the carboxyl group of L-cysteine, and that the 2-thiazoline-4-carboxylate moiety is derived from the second L-cysteine in the adult lantern of the firefly.

Materials and Methods

Chemicals

The stable isotope-labeled chemicals, L-[U-$^{13}C_3$]-cysteine (L-Cys[U-$^{13}C_3$]: 98% isotopic purity), L-[1-^{13}C]-cysteine (L-Cys[1-^{13}C]: 99% isotopic purity), L-[3-^{13}C]-cysteine (L-Cys[3-^{13}C]: 99% isotopic purity) and 1,4-[D_6]-hydroquinone ([D_6]-hydroquinone: 98% isotopic purity), were purchased from Cambridge Isotope Laboratories (Andover, MA), and the chemical purities of these compounds were over 98%. *p*-[2,3,5,6-D]-Benzoquinone ([D_4]-benzoquinone) was prepared from [D_6]-hydroquinone as previously reported [28]. In brief, the reaction mixture of [D_6]-hydroquinone (348 mg, 3.05 mmol) and Ag_2O (60.1 mg, 0.259 mmol) in methanol (6 mL) was stirred for 5 min, and a solution of 30% aq. H_2O_2 (0.75 mL, 7.5 mmol) in methanol (10 mL) was added dropwise and continuously stirred for 40 min at room temperature. The reaction mixture was diluted to 30 mL with H_2O and extracted twice with 30 mL of diethyl ether and evaporated. The yellow needle-like crystals (310 mg, 90.4% yield) of [D_4]-benzoquinone were obtained and were further purified by a silica gel column (Wakogel C-300, Wako Pure Chemicals, Osaka, Japan), eluted

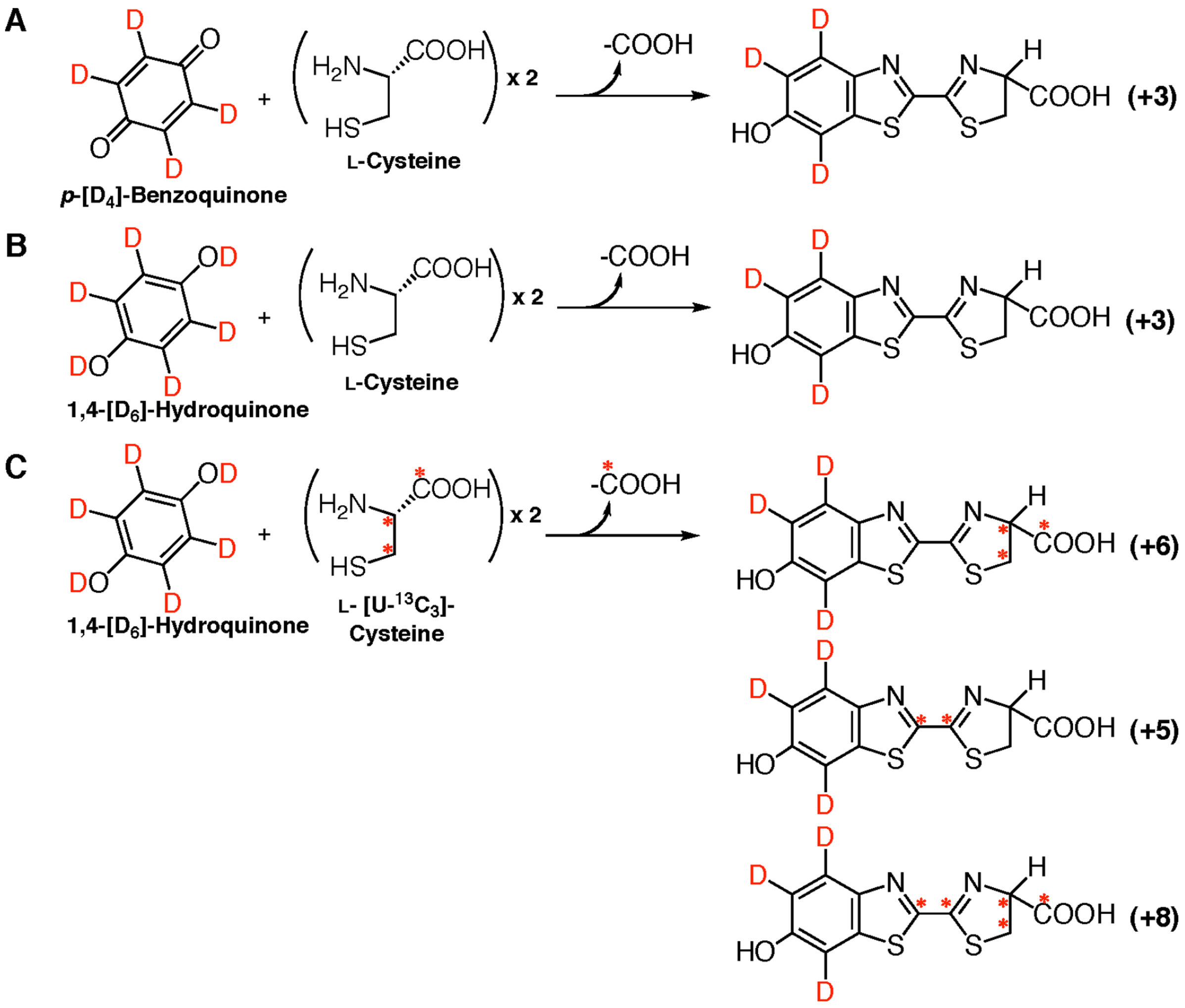

Figure 5. Incorporation of *p*-[D_4]-benzoquinone or 1,4-[D_6]-hydroquinone with L-cysteines into firefly luciferin in an adult lantern of *L. lateralis*. A. Predicted firefly luciferins incorporated from *p*-[D_4]-benzoquinone and L-cysteine. B. Predicted firefly luciferins incorporated from 1,4-[D_6]-hydroquinone and L-cysteine. C. Predicted firefly luciferins incorporated from 1,4-[D_6]-hydroquinone and L-Cys[3-$^{13}C_3$]. The number in parenthesis on the right indicates the number of the stable isotope atoms incorporated into firefly luciferin. Asterisk indicates the position of a ^{13}C-atom.
doi:10.1371/journal.pone.0084023.g005

Table 3. Relative isotopic peak intensities (%) of the parent and its fragment mass from firefly luciferin in the lantern extracts after injecting L-Cys[1-^{13}C] or L-Cys[3-^{13}C] with 1,4-hydroquinone into the adult of *L. lateralis*.

Number of stable isotope atom in MH^+	L-Cys[1-^{13}C] +1,4-hydroquinone			L-Cys[3-^{13}C] +1,4-hydroquinone		
	(*a*)[a]	(*b*)[a]	(*c*)[a]	(*a*)	(*b*)	(*c*)
+0	100.0	100.0	100.0	100.0	100.0	100.0
+1	**68.6**	16.3	10.9	**63.2**	**60.6**	**42.2**
+2	16.4	8.4	4.0	**42.7**	**43.3**	8.7
+3	5.8	1.3	–	8.4	10.0	–
+4	–	–	–	2.1	4.9	–
+5	–	–	–	–	–	–

[a](*a*) represents the parent mass of firefly luciferin with MH^+281 (+0, 100%). (*b*) and (*c*) represent the fragment ion mass from firefly luciferin with MH^+235 (+0, 100%) and MH^+177 (+0, 100%), respectively, as shown in Fig. 2. The numbers in bold are the significant mass peaks containing the incorporated stable isotope atoms.
doi:10.1371/journal.pone.0084023.t003

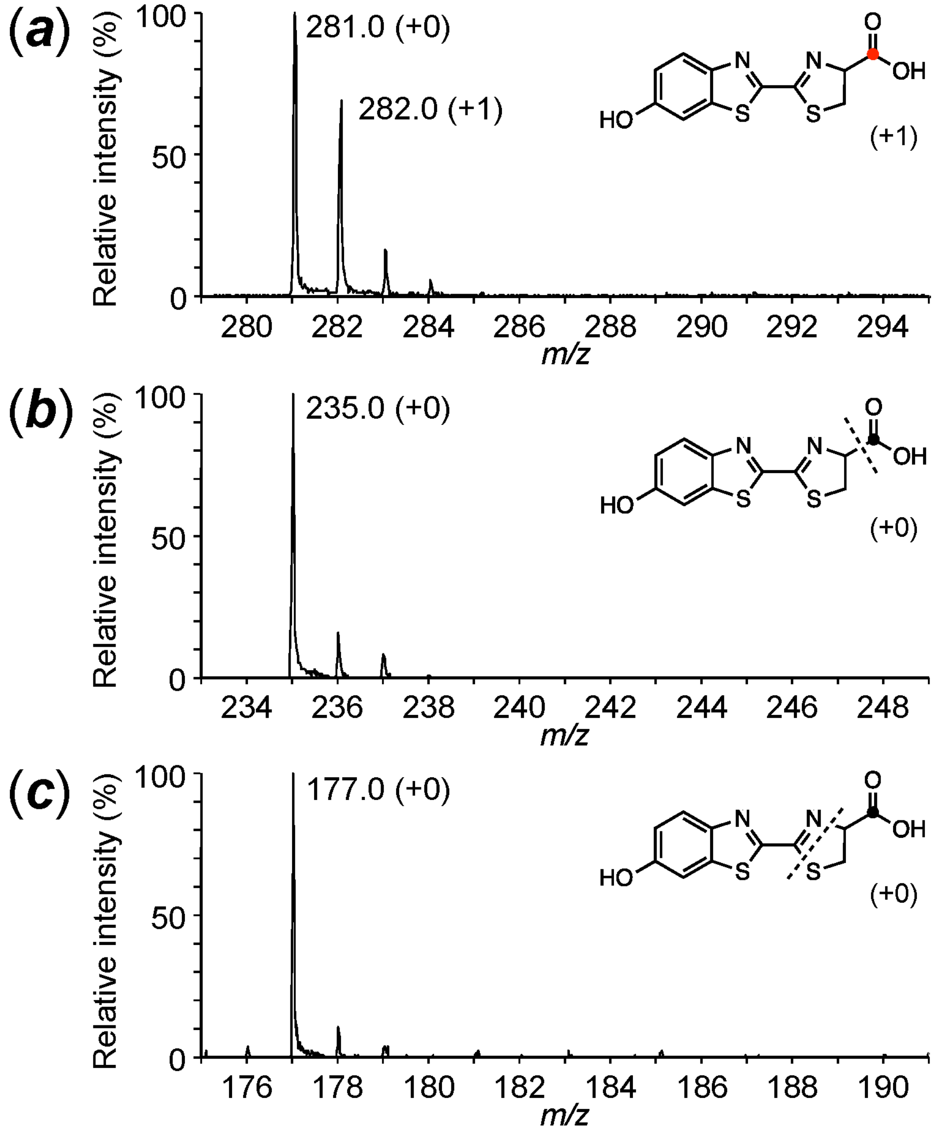

Figure 6. Mass spectra of firefly luciferin labeled with L-Cys[1-^{13}C] and 1,4-hydroquinone in the adult lantern of *L. lateralis*. (*a*), the parent ion; (*b*) and (*c*), the fragments ions of firefly luciferin.
doi:10.1371/journal.pone.0084023.g006

by *n*-hexane: ethyl acetate = 4:1. The chemical purity of [D_4]-benzoquinone on HPLC analysis was over 98%, and its isotopic purity was over 98% by ESI-TOF-MS analysis (negative mode) (data not shown). The non-isotopic chemicals were obtained from commercial sources: D-firefly luciferin potassium salt and arbutin (Wako Pure Chemicals); L-cysteine, D-cysteine and *p*-benzoquinone (Kanto Chemical, Tokyo, Japan); 1,4-hydroquinone (Nacalai Tesque, Kyoto, Japan). L-Firefly luciferin was kindly provided by Dr. Yoshiaki Toya (Aichi Univ. of Education, Aichi, Japan).

Specimens of Firefly, *Luciola Lateralis*

The adult specimens of Japanese firefly *Luciola lateralis* (Lampyridae, Coleoptera) reared in aquarium [29] were kindly provided by Mr. Haruyoshi Ikeya (Toin Gakuen High School, Yokohama, Japan).

Injection of Stable Isotope-labeled Compounds into the Adult Lantern of *L. lateralis*

The stock solutions of stable isotope-labeled and non-labeled compounds were prepared by dissolving compound in sterile H_2O to be 550 mM, excepting for 55 mM of *p*-benzoquinone and [D_4]-benzoquinone. For incorporation experiments, 1 μL of the stock solution was injected into the adult lantern of a female *L. lateralis* (within 4 days after adult emergence) using a microsyringe (701RN 10 μL SYR; Hamilton, Reno, NV) (Figure S13). After keeping fireflies in a moisture chamber at 24±2°C for 24 h, the living specimens injected were frozen in liquid nitrogen and stored at −80°C.

Extraction of the Labeled Firefly Luciferin from an Adult Lantern of *L. lateralis*

A single lantern was separated from a frozen specimen using a razor blade, and was homogenized in a tube with 70 μL of hot H_2O using a plastic pestle in a heating block at 95°C for 5 min to

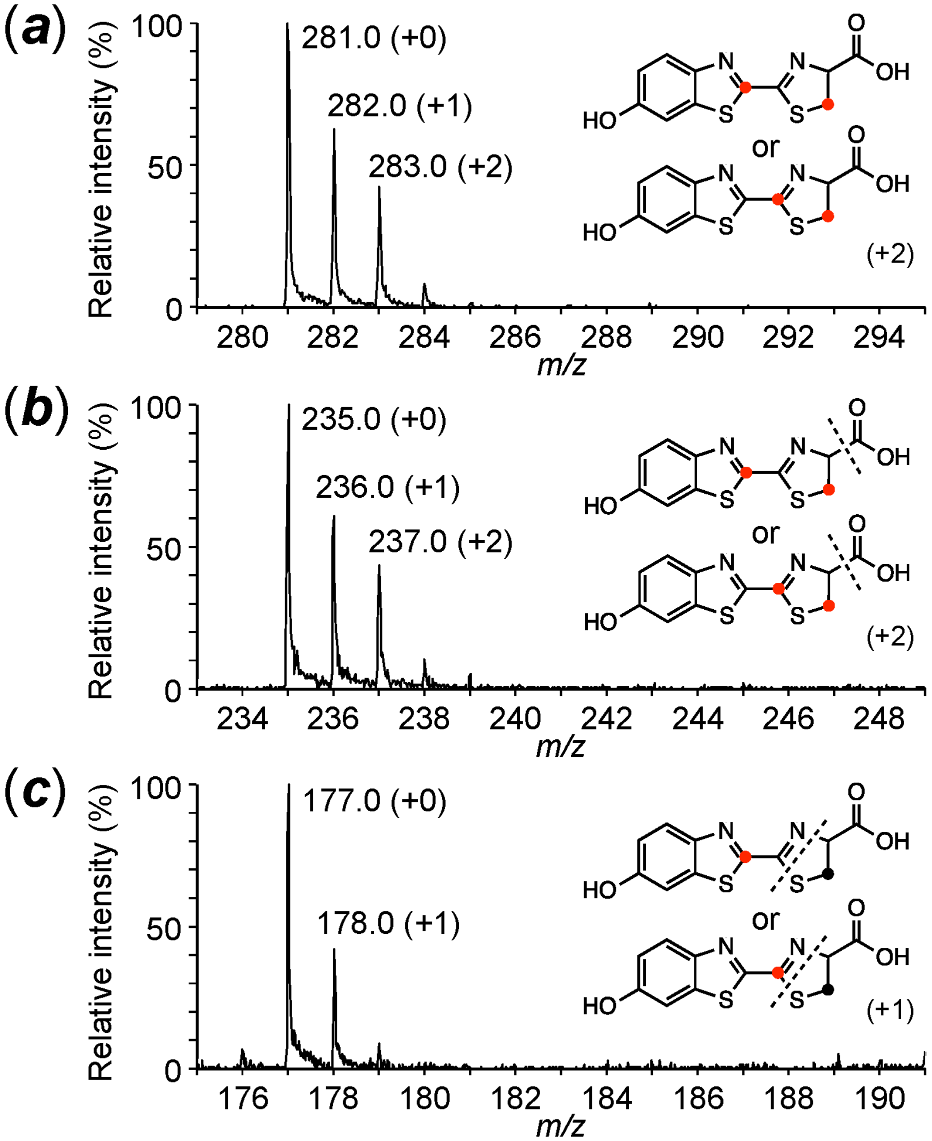

Figure 7. Mass spectra of firefly luciferin labeled with L-Cys[3-^{13}C] and 1,4-hydroquinone in the adult lantern of *L. lateralis*. (*a*), the parent ion; (*b*) and (*c*), the fragments ions of firefly luciferin.
doi:10.1371/journal.pone.0084023.g007

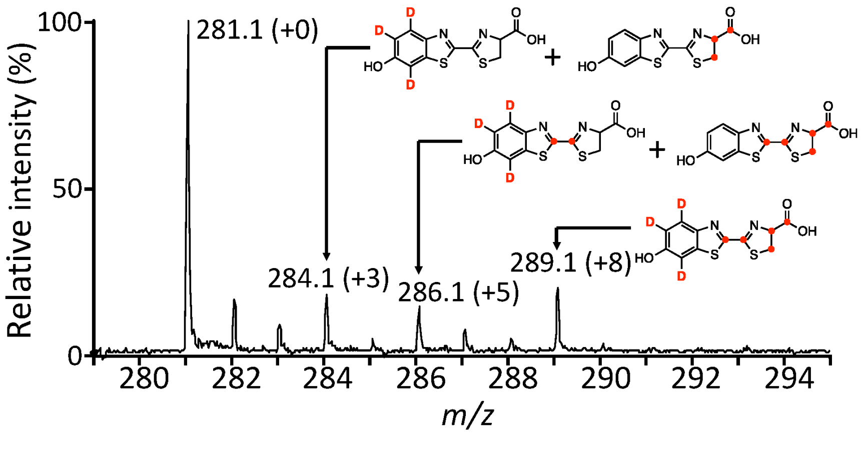

Figure 8. Mass spectrum of firefly luciferin labeled with L-Cys[U-^{13}C] and 1,4-[D_6]-hydroquinone into the adult lantern of *L. lateralis*.
doi:10.1371/journal.pone.0084023.g008

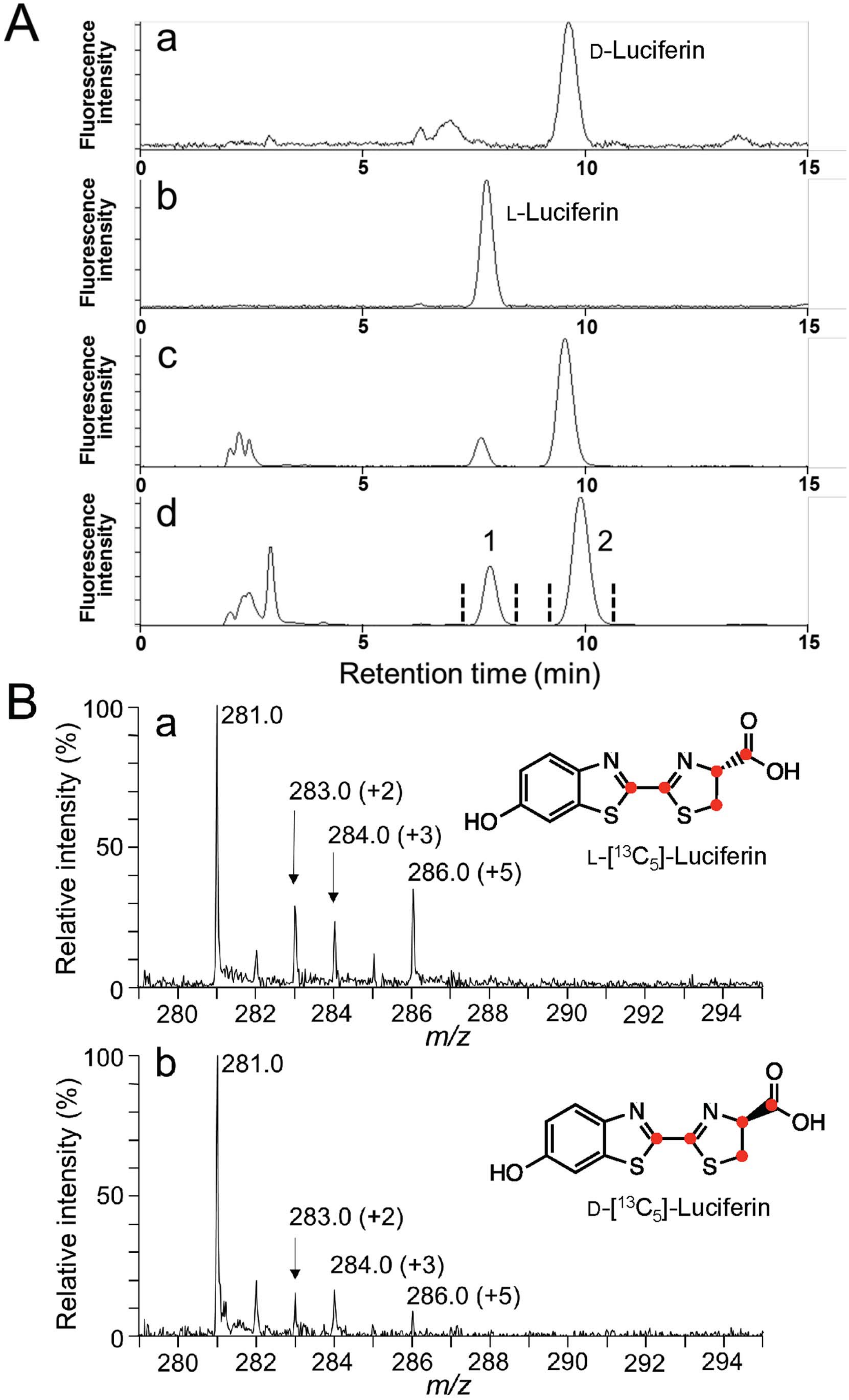
A
a
D-Luciferin
Fluorescence intensity
b
L-Luciferin
c
d
1
2
0
5
10
15
Retention time (min)
B
a
281.0
283.0 (+2)
284.0 (+3)
286.0 (+5)
L-[$^{13}C_5$]-Luciferin
Relative intensity (%)
m/z
b
281.0
283.0 (+2)
284.0 (+3)
286.0 (+5)
D-[$^{13}C_5$]-Luciferin
0
50
100
280
282
284
286
288
290
292
294

Figure 9. Identification of L- and D-luciferin in an adult lantern of *L. lateralis* by HPLC analysis and the incorporation of L-cysteine into D- and L-luciferin. A. Isolation of L- and D-firefly luciferin by HPLC analysis with a chiral column. (a) authentic D-luciferin, (b) authentic L-luciferin, (c) the extracts of *L. lateralis* lantern without injections, (d) the extracts of *L. lateralis* lantern after injection with L-Cys[U-$^{13}C_3$] and 1,4-hydroquinone. The peak fractions of 1 and 2, corresponding to L-luciferin and D-luciferin, respectively, are subjected to ESI-TOF-MS analysis, as shown in Fig. 9B. B. ESI-TOF-MS analyses of the HPLC fractions for L-luciferin and D-luciferin, separated by HPLC analysis as in Fig.9A–d. (a) L-luciferin separated from the lantern. (b) D-luciferin separated from the lantern.
doi:10.1371/journal.pone.0084023.g009

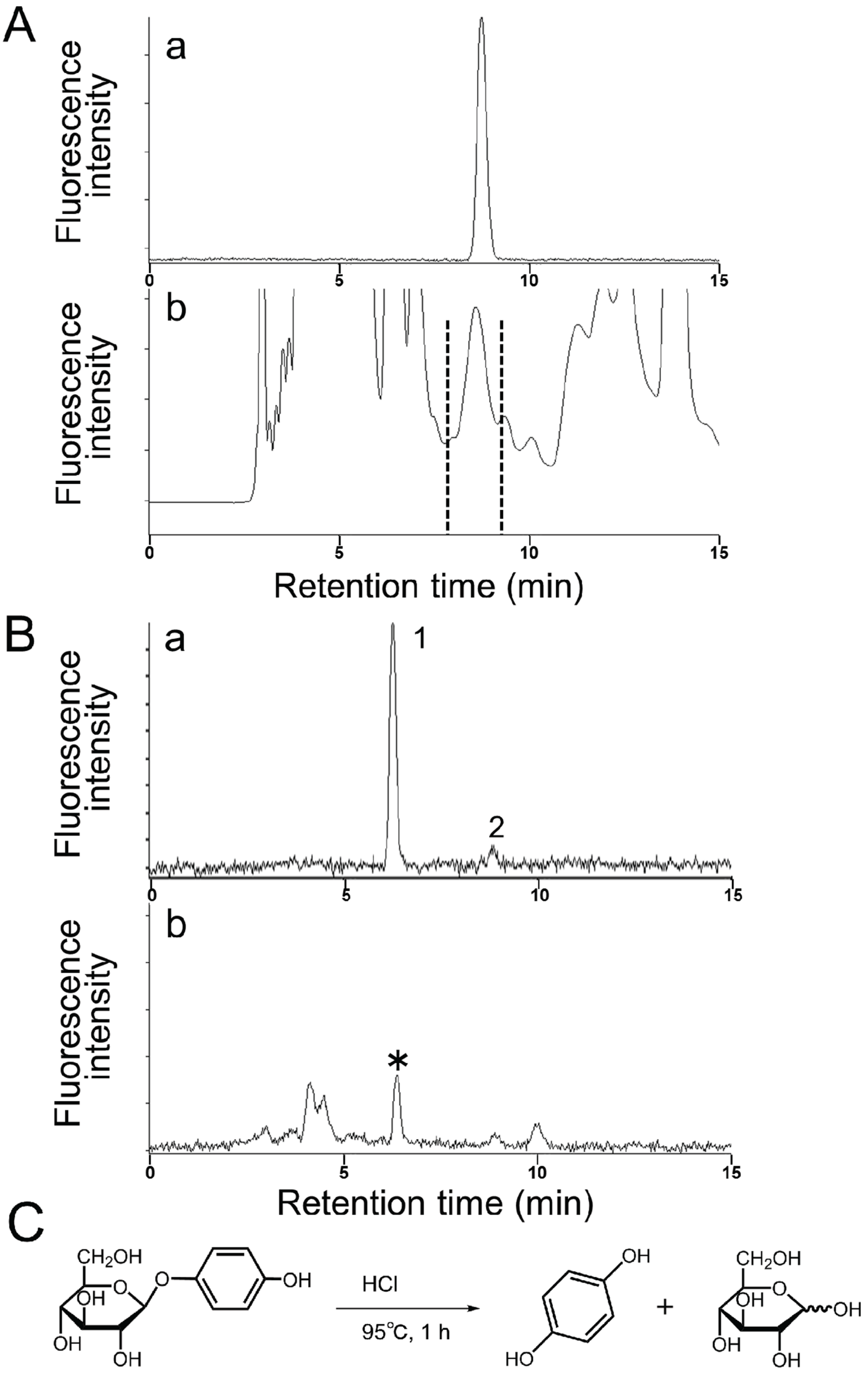

Figure 10. Identification of arbutin in *L. lateralis* by HPLC analysis. A. HPLC analysis of the extracts from an adult *L. lateralis* by using a fluorescence detector. (a) authentic arbutin, (b) the extracts of *L. lateralis* lantern. The arbutin fraction between the vertical dashed lines is used for hydrolysis as in Fig. 10C. B. HPLC analysis of the hydrolyzed arbutin fraction in Fig. 10A–b. (a) authentic 1,4-hydroquinone (labeled peak 1) containing benzoquinone (labeled peak 2), (b) the hydrolyzed products of the peak fraction between the dashed lines in Fig. 10A–b. Asterisk indicates 1,4-hydroquinone from arbutin. C. A scheme of acid hydrolysis of arbutin to 1,4-hydroquinone by acid treatment with HCl.
doi:10.1371/journal.pone.0084023.g010

inactivate luciferase activity. The homogenate was centrifuged at 17,400×g for 3 min at 4°C, and the resultant supernatant was filtrated by an Ultrafree-MC centrifugal filter (0.45 µm; Millipore, Billerica, MA). The filtrate obtained was washed twice by *n*-hexane (60 µL) and 5 µL of aqueous layer (ca. 30 µL) was subjected to LC/ESI-TOF-MS analysis. Under above extraction conditions at 95°C, the racemization of D/L-firefly luciferin could be partially occurred.

Identification of D- and L-luciferin in an Adult Lantern of *L. lateralis* by HPLC Analysis with a Chiral Column

To avoid racemization of D/L-firefly luciferin during the extraction from an adult lantern, a single lantern was homogenized in 70 µL of methanol at 4°C instead of hot H_2O at 95°C. Under above conditions, the racemization of D-luciferin to L-luciferin was not occurred at 4°C for 60 min. In contrast, by incubating D-luciferin in methanol at 70°C for 60 min, 21% of L-firefly luciferin was yielded by racemization (data not shown). Methanol extracts (18 µL) were analyzed by reversed-phase HPLC equipped with a chiral column, CHIRALCEL OD-RH (4.6×150 mm; Daicel Chemical Industry, Tokyo, Japan) and a fluorescence detector (FP-1520, Jasco). HPLC conditions: mobile phase, 27% acetonitrile in H_2O containing 0.1% formic acid; flow rate, 1.0 mL/min; excitation, 330 nm; emission, 530 nm. The eluted fractions containing D- or L-luciferin (1.2 mL) were collected, concentrated to ~20 µL using a rotary evaporator (N-N series, EYELA, Tokyo, Japan), and applied to LC/ESI-TOF-MS analysis.

LC/ESI-TOF-MS Analysis

LC/ESI-TOF-MS analysis was performed by electrospray ionization-ion trap-mass spectrometry (ESI-TOF-MS) on an Agilent 1100 HPLC system (Agilent Technologies, Santa Clara, CA) with a Mariner Biospectrometry Workstation (Applied Biosystems, Foster City, CA). HPLC conditions: column, Unison UK-C8 (75×2 mm; Imtakt, Kyoto, Japan); mobile phase, a linear gradient of methanol in H_2O containing 0.1% formic acid from 50% to 90% for 12 min; flow rate 0.1 mL/min; sprit ratio, 1:20 (5 µL/min); nozzle potential, 120–360 V; ion mode, positive. Under these conditions, the retention time of the mass ion peaks for D- and L-luciferin with its fragments was at approximately 5.5 min. The mass value was calibrated using angiotensin I (m/z = 324.9272 and 432.9603) and neurotensin (m/z = 558.3111) as external standards.

Isolation and Identification of Arbutin from *L. lateralis*

To isolate arbutin from *L. lateralis*, two frozen female adults were homogenized in 400 µL of methanol on ice. After centrifugation at 17,400×g for 3 min, the supernatant was filtrated by a 0.45 µm centrifugal filter. The filtrate was dried down under N_2 and suspended in 100 µL of 80% methanol. After incubating for 60 min on ice, the precipitate was removed by centrifugation at 17,400×g for 30 min. The resultant supernatant was dried down and dissolved in 50 µL of H_2O. The aqueous solution was washed three times with 50 µL of ethyl acetate, filtrated, and subjected to reversed-phase HPLC equipped with a Develosil ODS-UG-5 (4.6×250 mm; Nomura Chemical, Aichi, Japan) and a fluorescence detector (FP-1520, Jasco). HPLC conditions: mobile phase, 5% methanol in H_2O; flow rate, 1.0 mL/min; excitation, 280 nm; emission, 320 nm.

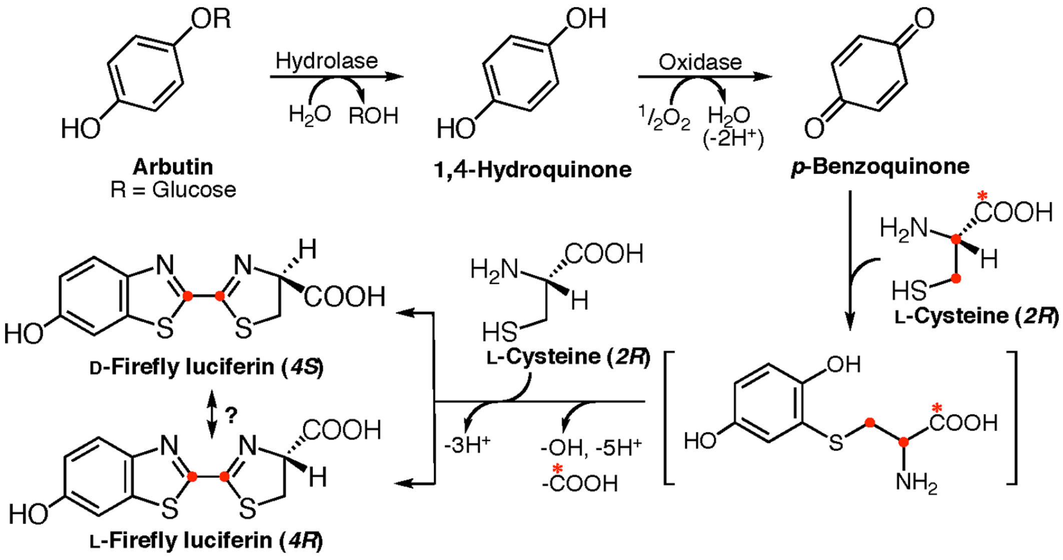

Figure 11. Proposed biosynthetic pathway of firefly luciferin in the lantern of adult firefly.
doi:10.1371/journal.pone.0084023.g011

To identify arbutin, the peak fraction (1.2 mL) containing arbutin was concentrated to 10 μL, and 5 μL was used for acid hydrolysis to release 1,4-hydroquinone from arbutin. The total reaction mixture (200 μL) containing 1.1 N HCl was incubated at 95°C for 1 h. After adding 200 μL of H_2O, the mixture was extracted three times with 400 μL of diethyl ether and the extracts were dried down under N_2. The resultant solid was immediately dissolved in 30 μL of H_2O and was analyzed by reversed-phase HPLC equipped with a Develosil ODS-UG-5 (4.6×250 mm) and a fluorescence detector. HPLC conditions: mobile phase, 25% methanol in H_2O; flow rate, 0.8 mL/min; excitation, 290 nm; emission, 338 nm.

To identify 1,4-hydroquinone in firefly, the hydrolyzed extracts obtained from 10 specimens were acetylated in 200 μL of acetic anhydride (Wako Pure Chemicals) and 1 μL of sulfuric acid (Wako Pure Chemicals) at room temperature for 5 min [30]. The acetylated products were subjected to LC/ESI-TOF-MS analysis as described above.

Supporting Information

Figure S1 Calculated mass spectra of firefly luciferin based on the natural isotopic abundance. (*a*), the parent ion of firefly luciferin; (*b*) and (*c*), the fragment ions of firefly luciferin.
(TIF)

Figure S2 Mass spectra of synthetic D-firefly luciferin. (*a*), the parent ion of firefly luciferin; (*b*) and (*c*), the fragment ions of firefly luciferin.
(TIF)

Figure S3 Mass spectra of synthetic L-firefly luciferin. (*a*), the parent ion of firefly luciferin; (*b*) and (*c*), the fragment ions of firefly luciferin.
(TIF)

Figure S4 Mass spectra of firefly luciferin extracted from an adult lantern of *L. lateralis* without injecting the labeled compounds. (*a*), the parent ion of firefly luciferin; (*b*) and (*c*), the fragment ions of firefly luciferin.
(TIF)

Figure S5 Injecting of L-Cys[U-$^{13}C_3$] into an adult lantern of *L. lateralis*. (*a*), the parent ion of firefly luciferin; (*b*) and (*c*), the fragment ions of firefly luciferin.
(TIF)

Figure S6 Injecting of L-Cys[U-$^{13}C_3$] and *p*-benzoquinone into an adult lantern of *L. lateralis*. (*a*), the parent ion of firefly luciferin; (*b*) and (*c*), the fragment ions of firefly luciferin.
(TIF)

Figure S7 Injecting of 1,4-[D_6]-hydroquinone into an adult lantern of *L. lateralis*. (*a*), the parent ion of firefly luciferin; (*b*) and (*c*), the fragment ions of firefly luciferin.
(TIF)

Figure S8 Injecting of 1,4-[D_6]-hydroquinone and L-Cys into an adult lantern of *L. lateralis*. (*a*), the parent ion of firefly luciferin; (*b*) and (*c*), the fragment ions of firefly luciferin.
(TIF)

Figure S9 Injecting of *p*-[D_4]-benzoquinone into an adult lantern of *L. lateralis*. (*a*), the parent ion of firefly luciferin; (*b*) and (*c*), the fragment ions of firefly luciferin.
(TIF)

Figure S10 Injecting of *p*-[D_4]-benzoquinone and L-Cys into an adult lantern of *L. lateralis*. (*a*), the parent ion of firefly luciferin; (*b*) and (*c*), the fragment ions of firefly luciferin.
(TIF)

Figure S11 Injecting of 1,4-[D_6]-hydroquinone and L-Cys[U-$^{13}C_3$] into an adult lantern of *L. lateralis*. (*a*), the parent ion of firefly luciferin; (*b*) and (*c*), the fragment ions of firefly luciferin.
(TIF)

Figure S12 Mass spectrum of acetylated 1,4-hydroquinone isolated from arbutin in *L. lateralis*.
(TIF)

Figure S13 Photograph of the procedure to inject the chemicals into the adult lantern of *L. lateralis* (female) using a syringe.
(TIF)

Table S1 Relative isotopic peak intensities (%) of calculated firefly luciferin, synthetic D-firefly luciferin and L-firefly luciferin.
(DOC)

Author Contributions

Conceived and designed the experiments: YO SI. Performed the experiments: YO NY SK. Analyzed the data: YO NY SK MO SI. Contributed reagents/materials/analysis tools: YO MO. Wrote the paper: YO SI. Obtained the living firefly: YO. Obtained L-luicferin: YO.

References

1. Shimomura O (2006) Bioluminescence: Chemical principles and methods. Singapore: World Scientific Publishing. 470 p.
2. Wilson T, Hastings JW (2013) Bioluminescence: Living lights, lights for living. Massachusetts: Harvard University Press. 185 p.
3. Lloyd JE (1978) Insect bioluminescence. In: Herring PJ, editor. Bioluminescence in action. London: Academic Press. 241–272.
4. Inouye S (2010) Firefly luciferase: An adenylate-forming enzyme for multicatalytic functions. Cell Mol Life Sci 67: 387–404.
5. White EH, McCapra F, Field GF, McElroy WD (1961) The structure and synthesis of firefly luciferin. J Am Chem Soc 83: 2402–2403.
6. White EH, McCapra F, Field GF (1963) The structure and synthesis of firefly luciferin. J Am Chem Soc 85: 337–343.
7. Seliger HH, McElroy WD, White EH, Field GF (1961) Stereospecificity and firefly bioluminescence, a comparison of natural and synthetic luciferins. Proc Natl Acad Sci U S A 47: 1129–1134.
8. Head JF, Inouye S, Teranishi K, Shimomura O (2000) The crystal structure of the photoprotein aequorin at 2.3 Å resolution. Nature 405: 372–376.
9. Oba Y, Kato S, Ojika M, Inouye S (2002) Biosynthesis of luciferin in the sea firefly, *Cypridina hilgendorfii*: L-tryptophan is a component in Cypridina luciferin. Tetrahedron Lett 43: 2389–2392.
10. Kato S, Oba Y, Ojika M, Inouye S (2004) Identification of the biosynthetic units of Cypridina luciferin in *Cypridina* (*Vargula*) *hilgendorfii* by LC/ESI-TOF-MS. Tetrahedron 60: 11427–11434.
11. Kato S, Oba Y, Ojika M, Inouye S (2006) Biosynthesis of Cypridina luciferin in *Cypridina noctiluca*. Heterocycles 72: 673–676.
12. Oba Y, Kato S, Ojika M, Inouye S (2009) Biosynthesis of coelenterazine in the deep-sea copepod, *Metridia pacifica*. Biochem Biophys Res Commun 390: 684–688.
13. Toya Y (2005) Biosynthesis of *Vargula hilgendorfii* luciferin, arisen from L-arginine, L-tryptophan, and L-isoleucine. In: Tsuji A, Matsumoto M, Maeda M, Kricka LJ, Stanley PE, editors. Proceedings of the 13th international symposium on bioluminescence & chemiluminescence. Singapore: World Scientific Publishing. 129–132.
14. Okada K, Iio H, Kubota I, Goto T (1974) Firefly bioluminescence III. Conversion of Oxyluciferin to luciferin in firefly. Tetrahedron Lett 15: 2771–2774.
15. Okada K, Iio H, Goto T (1976) Biosynthesis of firefly luciferin. Probable formation of benzothiazole form *p*-benzoquinone and cysteine. J Chem Soc Chem Commun: 32.
16. McCapra F, Razavi Z (1976) Biosynthesis of luciferin in *Pyrophorus pellucens*. J Chem Soc Chem Commun: 153–154.

17. Colepicolo P, Pagni D, Bechara EJH (1988) Luciferin biosynthesis in larval *Pyrearinus termitilluminans* (Coleoptera: Elateridae). Comp Biochem Physiol 91B: 143–147.
18. McCapra F, Razavi Z (1975) A model for firefly luciferin biosynthesis. J Chem Soc Chem Commun: 42–43.
19. Gomi K, Kajiyama N (2001) Oxyluciferin, a luminescence product of firefly luciferase, is enzymatically regenerated into luciferin. J Biol Chem 276: 36508–36513.
20. Niwa K, Nakamura M, Ohmiya Y (2006) Stereoisomeric bio-inversion key to biosynthesis of firefly D-luciferin. FEBS Lett 580: 5283–5287.
21. Oba Y, Shintani T, Nakamura T, Ojika M, Inouye S (2008) Determination of the luciferin contents in luminous and non-luminous beetles. Biochem Biocsci Biotechnol 72: 1384–1387.
22. Gnanasunderam C, Young H, Hutchins R (1985) Defensive secretions of New Zealand tenebrionids: V. Presence of methyl ketones in *Uloma tenebrionoides* (Coleoptera: Tenebrionidae). J Chem Ecol 11: 465–472.
23. Chadha MS, Eisner T, Meinwald J (1961) Defence mechanisms of arthropods-IV: *Para*-benzoquinones in the secretion of *Eleodes longicollis* Lec. (Coleoptera: Tenebrionidae). J Insect Physiol 7: 46–50.
24. Holliday AE, Walker FM, Brodie III ED, Formica VA (2009) Differences in defensive volatiles of the forked fungus beetle, *Bolitotherus cornutus*, living on two species of fungus. J Chem Ecol 35: 1302–1308.
25. Reinhard J, Lacey MJ, Ibarra F, Schroeder FC, Kaib M, Lenz M (2002) Hydroquinone: A general phagostimulating pheromone in termites. J Chem Ecol 28: 1–14.
26. Lembert N (1996) Firefly luciferase can use L-luciferin to produce light. Biochem J 317: 273–277.
27. da Silva LP, da Silva JC (2011) Kinetics of inhibition of firefly luciferase by dehydroluciferyl-coenzyme A, dehydroluciferin and L-luciferin. Photochem Photobiol Sci 10: 1039–1045.
28. Derikvand F, Bigi F, Maggi R, Piscopo CG, Sartori G (2010) Oxidation of hydroquinones to benzoquinones with hydrogen peroxide using catalytic amount of silver oxide under batch and continuous-flow conditions. J Catalysis 271: 99–103.
29. Oba Y, Furuhashi M, Bessho M, Sagawa S, Ikeya H, et al. (2013) Bioluminescence of a firefly pupa: involvement of a luciferase isotype in the dim glow of pupae and eggs in the Japanese firefly, *Luciola lateralis*. Photochem Photobiol Sci 12: 854–863.
30. Zhao W, Sun J, Xiang H, Zeng YY, Li XB, et al. (2011) Synthesis and biological evaluation of new flavonoid fatty acid esters with anti-adipogenic and enhancing glucose consumption activities. Bioorg Med Chem 19: 3192–3203.
